# TSG-6 promotes Cancer Cell aggressiveness in a CD44-Dependent Manner and Reprograms Normal Fibroblasts to create a Pro-metastatic Microenvironment in Colorectal Cancer

**DOI:** 10.7150/ijbs.69178

**Published:** 2022-02-07

**Authors:** Binbin Liu, Tengfei Liu, Yiting Liu, Xingzhi Feng, Xuefei Jiang, Jiahui Long, Shubiao Ye, Daici Chen, Jianping Wang, Zihuan Yang

**Affiliations:** Guangdong Provincial Key Laboratory of Colorectal and Pelvic Floor Diseases, Guangdong Institute of Gastroenterology, the Sixth Affiliated Hospital of Sun Yat-sen University, Guangzhou, Guangdong 510655, China.

**Keywords:** TSG-6, Fibroblast, Colorectal Cancer, Metastasis

## Abstract

Tumor necrosis factor α stimulated gene 6 (TSG-6), a 30-KD secretory protein, plays an essential role in modulating inflammatory responses and extracellular matrix remodeling. However, little is known regarding the role of TSG-6 in human cancers. Here, we investigated the mechanism of action and the role of TSG-6 in colorectal cancer (CRC) metastasis. We found that TSG-6 was highly expressed in tumor tissues and was associated with poor prognosis and metastasis in CRC. Mechanistically, TSG-6 overexpression in CRC cells resulted in ERK activation and epithelial-mesenchymal transition by means of stabilizing CD44 and facilitating the CD44-EGFR complex formation on the cell membrane. Consequently, this resulted in the promotion of tumor migration and invasion both *in vitro* and *in vivo*. Notably, our data showed that CRC cells secreted TSG-6 could trigger a paracrine activation of JAK2-STAT3 signaling and reprogram normal fibroblasts into cancer-associated fibroblasts, which exhibited upregulation of pro-metastatic cytokines (CCL5 and MMP3) and higher movement ability. In animal models, the co-injection of cancer cells and TSG6-reprogrammed fibroblasts led to a significant increase in tumor metastasis. Our findings indicated that TSG-6 overexpression in CRC cells could promote cancer metastasis in both an autocrine and paracrine manner. Therefore, targeting TSG-6 might be a potential therapeutic strategy for the treatment of metastatic CRC.

## Introduction

Colorectal cancer (CRC) is the third most commonly diagnosed cancer and the second leading causes of cancer-related mortality worldwide 1, 2. While metastasis is the leading cause of death in CRC patients. Locally advanced CRC patients are typically treated with chemotherapy to prevent tumor metastasis. However, approximately 30% of these patients will finally develop metastases and die 3. Furthermore, the 5-year overall survival rate of CRC patients presenting with distant metastasis at the time of first diagnosis is only 14% 2. Therefore, there is a vital need to identify effective therapeutic strategies to combat tumor metastasis.

Consensus molecular subtypes (CMS) is a CRC classification system based on molecular alterations such as genomic drivers, transcriptomic subtypes and immune signatures 4, 5. Currently, there are four CMS, each associated with distinct CRC features. Notably, CMS4 tumors are characterized by epithelial-mesenchymal transition (EMT), significant fibroblast and immune cell infiltration, and extracellular matrix (ECM) remodeling 4, 5. Patients with “mesenchymal” CMS4 tumors have the worst survival rate compared with other CMS. EMT is a cellular process triggered by signals that cells receive from the tumor microenvironment (TME), during which epithelial cells lose their epithelial features and gain mesenchymal phenotypes 6. The role of EMT in cancer metastasis has been extensively studied. However, EMT contribution to stromal cell infiltration is not fully understood.

The interaction between cancer cells and TME is crucial in regulating cancer development 7. As the principal stromal cells in the ECM, normal fibroblasts (NFs), under normal conditions, are considered to play an onco-suppressive role. Conversely, in established tumors, cancer cells can exploit NFs by direct cell-cell contact or indirect communication, thereby converting them into protumoral cancer-associated fibroblasts (CAFs) 8, 9. Unraveling the potential interaction between cancer cells and fibroblasts may help in developing novel therapeutic strategies.

Tumor necrosis factor-α (TNF-α) stimulated gene 6 (TSG-6) is a secreted protein and a member of the hyaluronan-binding protein family 10. Even though the role of TSG-6 in inflammation has been extensively researched, its function and underlying mechanism in cancer are rarely studied 11-13. As inflammation is a critical component of tumor progression, it is worth investigating the impact of TSG-6 on cancer progression. The TSG-6 hydrophobic Link module can bind to the main ECM component, hyaluronan (HA), which induces a HA conformational change and ECM reorganization 14. The HA-binding protein CD44, a cell surface marker for predicting metastasis and progression in various cancer types, has been reported to be associated with TSG-6 15, 16.

In the present study, we found that the TSG-6 level is significantly higher in CMS4 CRC, which is associated with the worst survival outcome. TSG-6 overexpression in CRC tumor areas is significantly associated with poor prognosis and metastasis. Moreover, TSG-6 could enhance the interaction between CD44 and EGFR, subsequently activating downstream ERK signaling and EMT. Furthermore, TSG-6 could trigger the paracrine activation of JAK2-STAT3 signaling thereby converting NFs into CAFs. The CAFs secrete cytokines, including CCL5 and matrix metalloproteinases (MMPs), to further promote cancer metastasis. Accordingly, TSG-6 is considered a potential novel therapeutic target for metastatic CRC.

## Materials and Methods

### Reagents and vectors

Primary antibodies used in western blot, immunofluorescence, immunohistochemistry (IHC), and neutralizing antibody analysis are listed in Supplementary Table 1. The pcDNA 3.1(+) plasmid was purchased from Invitrogen (Carlsbad, CA, USA). The lentiviral expression vector pCDH-CMV-MCS-EF1-copGFP was acquired from SBI Pharmaceuticals (Tokyo, Japan). The STAT3 inhibitor was obtained from Selleck Chemical (Houston, TX, USA).

### Patient Samples

A total of 39 pairs of normal (a distance of 5cm from the tumor border), paratumor (a distance of 2 cm from the tumor border) and tumor tissues were obtained from CRC patients at the Sixth Affiliated Hospital of Sun Yat-sen University (SYSU), China, and approved by the Human Medical Ethics Committee of the Sixth Affiliated Hospital of SYSU. Subsequently, primary CRC tissue microarrays (TMA) from 206 patients from our hospital were constructed (SYSU-cohort). None of the patients included in the study had received adjuvant chemotherapy or radiotherapy.

### Cell Lines

CRC cells (HCT116, DLD1) were purchased from the American Type Culture Collection (ATCC) and cultured according to the ATCC guidelines. Cells were allowed to grow in a humidified incubator at 37 °C with 5% CO2.

### Plasmid Constructs, siRNAs and Transfections

For the TSG-6 transient transfection, the full length of the human *TSG-6* cDNA open reading frame (ORF) was cloned into the pcDNA 3.1(+) plasmid using an In-fusion HD Cloning Kit (Clonetech, Tokyo, Japan). Control vector and pcDNA 3.1-TSG-6 plasmids were then transfected into CRC cells with Lipofectamine® 3000 (Invitrogen) according to manufacturer's instructions. The gene encoding mCherry protein was cloned into the control vector and pcDNA 3.1-TSG-6 plasmids to produce a corresponding mCherry labeled plasmid.

To ensure stable transfection, the full length of the human *TSG-6* cDNA ORF was cloned into the lentiviral expression vector pCDH-CMV-MCS-EF1-copGFP (SBI Pharmaceuticals, Tokyo, Japan). HEK 293T cells were co-transfected with the pCDH-TSG-6/pCDH-Vector, pCMV-Δ8.91 and pCMV-VSVG using Lipofectamine® 3000 (Invitrogen) to produce the lentivirus. The culture medium was replaced 24h after transfection and the virus supernatant was collected at 36h, 48h, 72h post-transfection. Luciferase-expressing HCT116 cells were infected with the amassed virus. Infected cells were then treated with polybrene and selected with puromycin for two weeks. Subsequently, all plasmid constructs were confirmed by sequencing. The primers used in plasmid construction are listed in Supplementary Table 2. The TSG-6 stable overexpressed HCT116 cells were only used in animal experiments in this study.

Small interfering RNA for TSG-6 and CD44 was purchased from RiboBio (Guangzhou, China). CRC cells were transfected with siRNA using Lipofectamine^TM^ RNAiMAX (Invitrogen). Following 48h of transfection, transfected cells were employed in RT-qPCR, western blot, and the migration/invasion assay.

### Isolation of Primary Fibroblasts

Fibroblasts were isolated as described elsewhere 17. In brief, normal colon tissues (a minimum of 10cm away from the primary tumor site) were collected from CRC patients who did not receive adjuvant radiotherapy or chemotherapy. Colon tissues were minced and digested in collagenase type IV (Sigma Aldrich, Germany) at 37 °C for 1h. Digested cell mixtures were cultured in MEM medium with 10% fetal bovine serum (FBS) and sodium pyruvate in a humidified atmosphere containing 5% CO2 for 4-6 hours. Subsequently, the supernatant was replaced to exclude all unattached cells. The NFs used in the experiments were within 15 passages. Moreover, NFs isolated from five different patients were pooled together for use in all subsequent studies and to avoid individual discrepancies.

### Coculture assay

mCherry-labeled HCT116 cells transfected with control vector or TSG-6 overexpressing plasmids were cocultured in a 12-well plate with NFs infected with green fluorescent protein (GFP) expressing lentiviral vector. Cocultures were then monitored using the High Content Screening System equipment (Operetta CLS, PerkinElmer).

### Wound healing assay and cell movement analysis

The Ibidi^®^ Culture Insert chamber (Ibidi®, German) was set onto a 24mm pre-coated plastic dish (Thermo Fisher Scientific). 100µl cell suspensions with 1.0×10^5^ fibroblasts were added to each well of the chamber and incubated at 37°C and 5% CO_2_. Cells were removed once full confluence attained in each well. Floating cells were washed with phosphate buffer saline (PBS) before adding culture medium containing 1% FBS. The wound healing process was captured from immediately after scratches to 36h later by the Incucyte Zoom System (Essen Bioscience, USA), photographed every two hours. The wound coverage area was measured using ImageJ software. Each assay had three biological repeats.

For cell movement and morphology analysis, fibroblasts were plated in 12-well plates and dyed with CellTracker^TM^ Blue CMAC (ThermoFisher Scientific, USA). Moreover, F-actin was stained with phalloidin and then labeled nuclei with Hoechst. Cell movement and morphology analysis were performed and the average cell size, F-actin intensity, movement speed, and fibroblasts accumulated distance were calculated using High-Content Imaging System equipment (Operetta CLS, PerkinElmer).

### Human cytokine antibody arrays

Fibroblasts were treated for 72h with recombinant human TSG-6 (rhTSG-6) or conditioned medium from TSG-6-overexpressed HCT116 cells. Next, the fibroblasts' culture supernatant of fibroblasts was collected and detected using Human Cytokine Antibody Arrays 440 Kit (GSH-CAA-440, Raybiotech) according to the manufacturer's instructions. Cytokines with a signal intensity > 1000 and a fold change > 2 were selected for further analysis.

### RNA sequencing and bioinformatic analysis

Total RNA from fibroblasts with or without a 72h rhTSG-6 treatment was isolated using TRIzol reagent (Ambion, CA, USA). Whole transcriptome sequencing was performed by the Beijing Genomics Institute (BGI-tech) on the BGISEQ-500 platform. Altered genes with heat maps were analyzed by employing the Dr. Tom platform (BGI-tech, China), Gene Ontology (GO), and Kyoto Encyclopedia of Genes and Genomes (KEGG). Collectively, these databases were used to identify the critical biological pathways regulated by rhTSG-6 treatment in fibroblasts. Gene Set Enrichment Analysis (GSEA) was conducted using the GSEA preranked tool downloaded from the GSEA website (http://software.broadinstitute.org/GSEA/msigdb/annotate.jsp) or with R studio.

### TMA Construction and Immunohistochemistry

Paraffin-embedded CRC tissue blocks from the Sixth Affiliated Hospital of SYSU were used to construct TMA slides, as described in our previous study 18. Immunohistochemistry (IHC) analysis was performed using the standard protocol. Briefly, the paraffin sections were placed in a dry oven at 60 °C overnight and then deparaffinized in xylene three times and rehydrated in graded ethanol. The sections were soaked in Tris-EDTA-containing (pH 8.0) distilled water and boiled for 15min to achieve antigen retrieval. Until the water cooled down naturally, the sections were taken out and incubated in 0.3% H_2_O_2_ for 10 min to block endogenous peroxidase activity. The sections were blocked with goat serum for 1h and subsequently incubated with a specific primary antibody overnight at 4 °C. Next, the sections were stained with diaminobenzidine and counterstained with hematoxylin. Subsequently, two pathologists independently evaluated the immunostaining of the sections. To evaluate the TSG-6 protein level, each section was assigned a score based on both the staining area and intensity. The intensity was scored from 0 to 3 (0 indicating no staining, 1 indicating weak staining, 2 indicating moderate staining, 3 indicating strong staining). The scores were based on the percentage of positive cancer cells and are, therefore defined as follows: 1 (0-25% of positive cells), 2 (26-50% of positive cells), 3 (51-75% of positive cells), and 4 (76-100% of positive cells). To generate a final score, we multiplied the two scores together yielding a final score ranging from 0 to 12. The tissue samples were then ranked by their final score. Finally, X-tile was used to establish the optimal cutoff point for dividing samples into two groups: samples with scores>4 defined as TSG-6 high expression, scores≤4 defined as TSG-6 low expression.

### Animal Experiments

Female BALB/C nude mice, four to five weeks old, were purchased from VitalRiver Laboratory Animal Technology (Beijing, China). The mice were raised in pathogen free conditions at the Experimental Animal Center of the Sixth Affiliated Hospital of Sun Yat-sen University.

To evaluate the function of TSG-6 in CRC liver metastasis *in vivo*, intrasplenic injection of luciferase-labeled HCT116 cells were employed in this study. 5.0×10^5^ cells (HCT116^Vector^, HCT116^TSG-6^) in 50μl sterile PBS were injected into the mouse spleen with or without 2.0×10^5^ fibroblasts. Seven weeks after injection, the mice were sacrificed and the metastatic foci on the liver surface were counted.

To evaluate whether TSG-6-activated fibroblasts could promote CRC liver metastasis *in vivo*, luciferase-labeled HCT116 cells (5.0×10^5^ cells) and fibroblasts (2.0×10^5^ cells) pretreated with rhTSG-6 for 24h were coinjected into the mouse spleen. Four weeks after injection, the mice were sacrificed and the metastatic foci on the liver surface were counted. The bioluminescence imaging (BLI) signals were monitored weekly using an *in vivo* imaging system (IVIS) (PerkinElmer, MA, USA). The livers were fixed with 4% paraformaldehyde and then embedded in paraffin for further analysis (IHC, Sirius Red staining, H&E staining).

### Statistical Analysis

All experiments were repeated at least three times. All data are shown as mean± standard error of mean (S.E.M). Statistical analysis was performed using SPSS 22.0 (Chicago, IL, USA) and GraphPad Prism 8.0 (La Jolla, CA, USA). The Kaplan-Meier and log-rank tests were conducted for survival analysis. The χ2 test was used to analyze the clinicopathological data's qualitative variables. Wilcoxon tests were used to compare TSG-6 mRNA and protein levels between tumor and normal tissues. Univariable and multivariable Cox proportional hazards regression were utilized to assess the CRC patient's prognostic factors. A two-tailed Student's *t*-test or one-way ANOVA was performed to evaluate the significant difference between the two groups. *P* < 0.05 was considered statistically significant.

### Additional Materials and Methods

Additional materials and methods can be found in the Supplementary Information.

## Results

### TSG-6 overexpression is correlated with poor clinical outcomes in CRC patients

**Figure 1:**
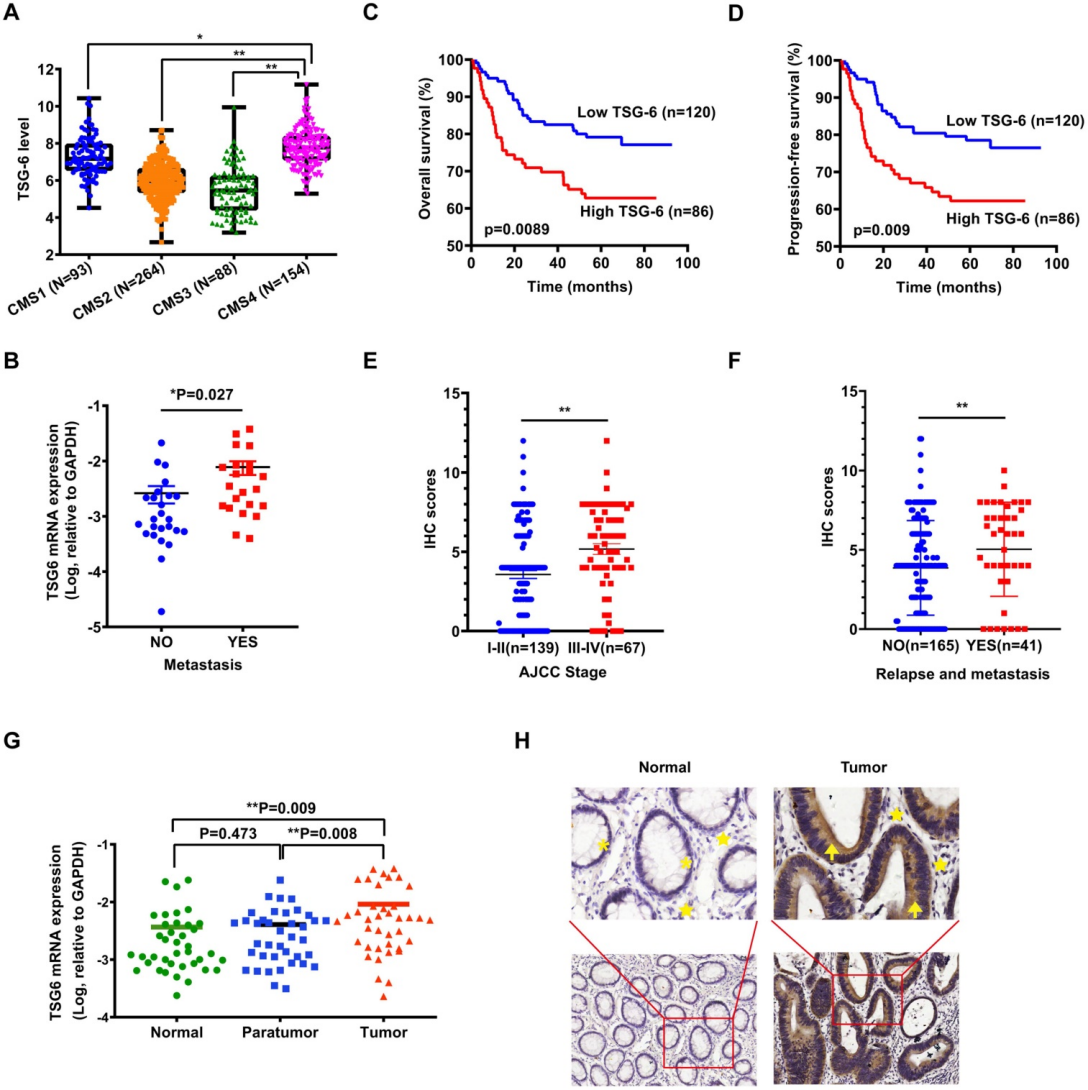
** TSG-6 is upregulated in CRC and associated with poor prognosis. (A)** TSG-6 mRNA level in TCGA CRC tumors separated by CMS. **(B)** Quantification of TSG-6 mRNA in CRC patients with metastasis (n=25) and without metastasis (n=22). **(C-D)** OS (C) and PFS (D) of CRC patients (SYSU-cohort), stratified by TSG-6 IHC-score. **(E)** Quantification of TSG-6 IHC score in CRC patients (SYSU-cohort) separated by Stage I-II and Stage III-IV. **(F)** Quantification of TSG-6 IHC score in CRC patients (SYSU-cohort) separated by metastasis status. **(G)** Quantification of TSG-6 mRNA level in CRC tumor tissues, paired paratumor and normal tissues (n=39). **(H)** The representative images of immunohistochemistry staining of TSG-6 in tumor tissues and paired adjacent normal tissues. Yellow asterisks: normal epithelial cells. Yellow stars: stromal cells. Yellow arrows: cancer cells. The boxes in (A) indicate interquartile range, horizontal black lines represent median. Error bars in (B), (E), (F) and (G) represent mean ± S.E.M. Statistical analysis was performed using One-way ANOVA test (A), Log-rank Gehan-Breslow-Wilcoxon test (C, D), two tailed unpaired Student's t-test (B, E, F) and paired t-test (G). *p<0.05, **p<0.01.

As reported, transcriptional profiling has identified four CRC consensus molecular subtypes (CMS) depicting distinct prognostic profiles 5. To investigate the possible role of TSG-6 in CRC, we first evaluated whether TSG-6 is correlated with clinical outcome using The Cancer Genome Atlas (TCGA) datasets. Interestingly, we established that a high TSG-6 mRNA level was significantly associated with CMS4 tumors, which are usually diagnosed at more advanced CRC stages and are associated with more aggressive and metastatic tumors compared to the other CMS (Figure 1A) 4, 19. Next, we detected TSG-6 mRNA levels in 47 fresh frozen primary tumor samples and found that TSG-6 levels were significantly higher in metastatic CRC (n=22) compared with non-metastatic CRC (n=25) (*P* = 0.027, Figure 1B). Immunohistochemistry (IHC) examination on tumor tissue microarray of our own CRC cohort (SYSU-cohort) showed that TSG-6 was positively stained in tumor areas in most CRC patients (Figure S1A). Kaplan-Meier analysis demonstrated that high TSG-6 protein expression correlated with poor overall survival (OS) (Figure 1C) and progression-free survival (PFS) (Figure 1D). A high TSG-6 IHC score is significantly associated with a higher TNM stage (Figure 1E) and metastasis (Figure 1F and Table 1). Univariate and multivariate Cox regression analysis demonstrated that TSG-6 was not an independent predictor for OS and PFS (Tables 2 & 3), but was a significant indicator of progression and metastasis. Consistently, survival analysis using the TCGA dataset showed that high TSG-6 mRNA expression levels correlated with poor disease specific survival (DSS) (Figure S1B,* P* = 0.048). As TSG-6 is significantly overexpressed in tumor tissues compared with paired normal or paratumor tissues (Figure 1G and 1H), and its upregulation was primarily observed in cancer cells (Figure 1H and S1C-S1D), the findings indicated that TSG-6 might act as an oncogenic marker in cancer cells associated with poor prognosis and tumor metastasis in CRC.

### TSG-6 promotes migration and invasion in CRC cells by inducing EMT

**Figure 2:**
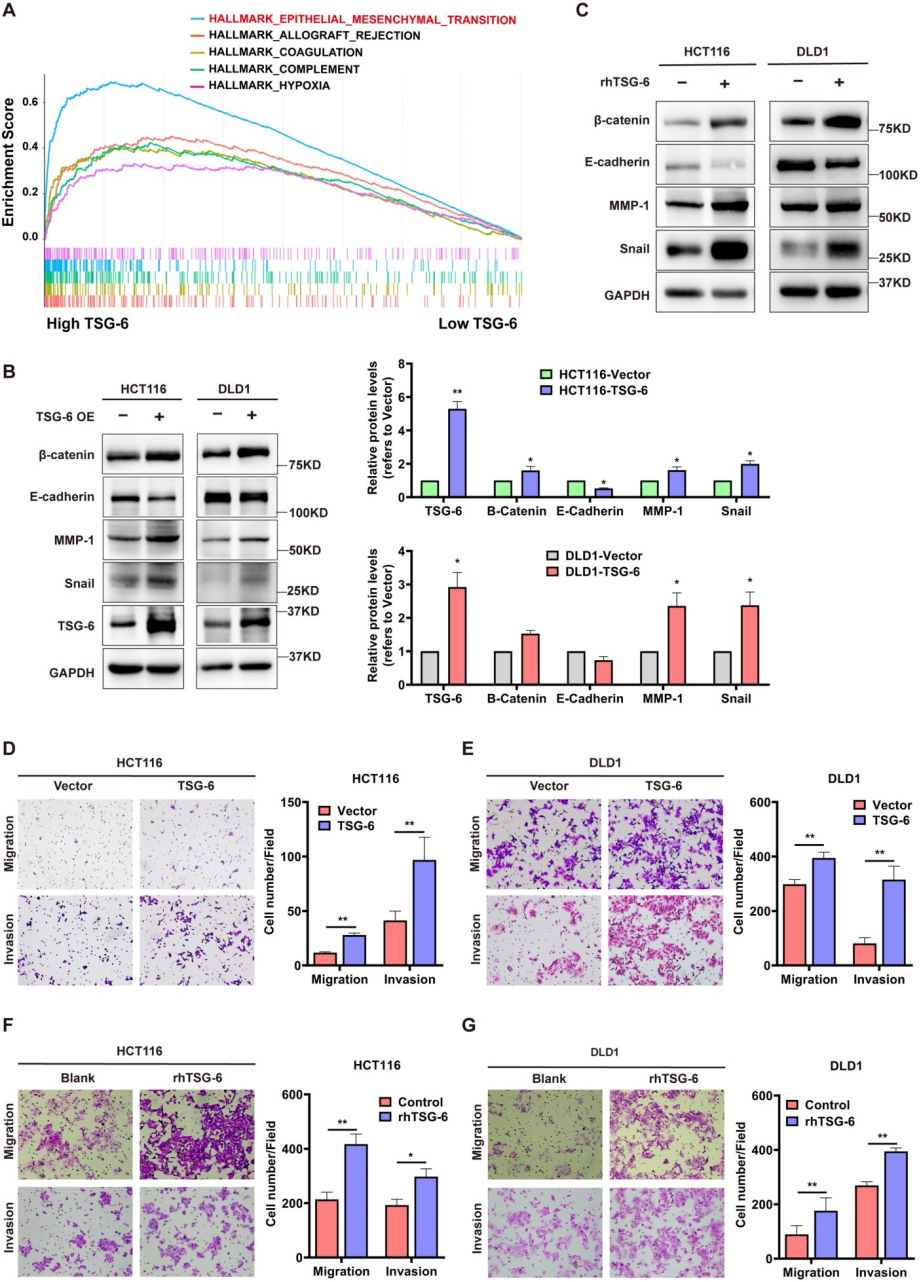
** TSG-6 promotes migration and invasion and induces EMT in CRC cells. (A)** GSEA results showing HALLMARK (epithelial-mesenchymal transition) using GSE14333 datasets. **(B-C)** Western blot of indicated EMT markers in CRC cells transiently overexpressing TSG-6 (B) or treated with rhTSG-6 (C). **(D-G)** Transwell migration and invasion assays performed in CRC cells transiently overexpressing TSG-6 (D and E), or treated with rhTSG-6 (F and G). Cell numbers shown in the bar chart were the average of five random fields. Data information: Error bars represent mean ± S.E.M. Statistical analysis was performed using two tailed unpaired Student's t-test (B, D, E, F, G). *p<0.05, **p<0.01.

Next, we performed Gene Set Enrichment Analysis (GSEA) in CRC (GEO: GSE14333, n=290) to identify TSG-6 functional associations. GSEA showed that a high TSG-6 level was mainly associated with ECM remodeling, especially in relation to collagen activity, cytokine, and chemokine activity (Figure S2A). Notably, EMT gene sets were enriched in samples with high TSG-6 levels (Figure 2A), in line with the CMS4 tumors that were characterized by the mesenchymal phenotype and the upregulation of EMT-associated genes 4.

Considering this observation, we sought to investigate the *in vitro* function of TSG-6 in CRC. First, we established TSG-6 overexpressed CRC cell lines via transient plasmid transfection (HCT116 and DLD1) and observed the TSG-6 mRNA level in the cell lysate and supernatant protein level were dramatically increased in TSG-6 overexpressed cells compared with control cells (Figure S2B-S2D). Consistent with GSEA analysis, TSG-6 overexpression in CRC cells exhibited the EMT phenotype indicated by the increase of β-catenin, snail, and MMPs, along with reduced E-cadherin expression (Figure 2B and S2E). Similar results were obtained from CRC cells treated with 100 ng/ml recombinant human TSG-6 (rhTSG-6) (Figure 2C). Either TSG-6 overexpression or rhTSG-6 application significantly promoted cell migration and invasion in CRC cells (Figure 2D-G), however, this did not affect proliferation (Figure S2F). Moreover, TSG-6 downregulation could suppress CRC cells' migration and invasion abilities (Figure S2G and S2H). Accordingly, these findings indicate that TSG-6 overexpression in CRC cells could induce migration and invasion depending on EMT and MMPs release.

### TSG-6 promotes CRC cell metastasis by facilitating cell membrane CD44-EGFR complex formation and downstream ERK activation

**Figure 3:**
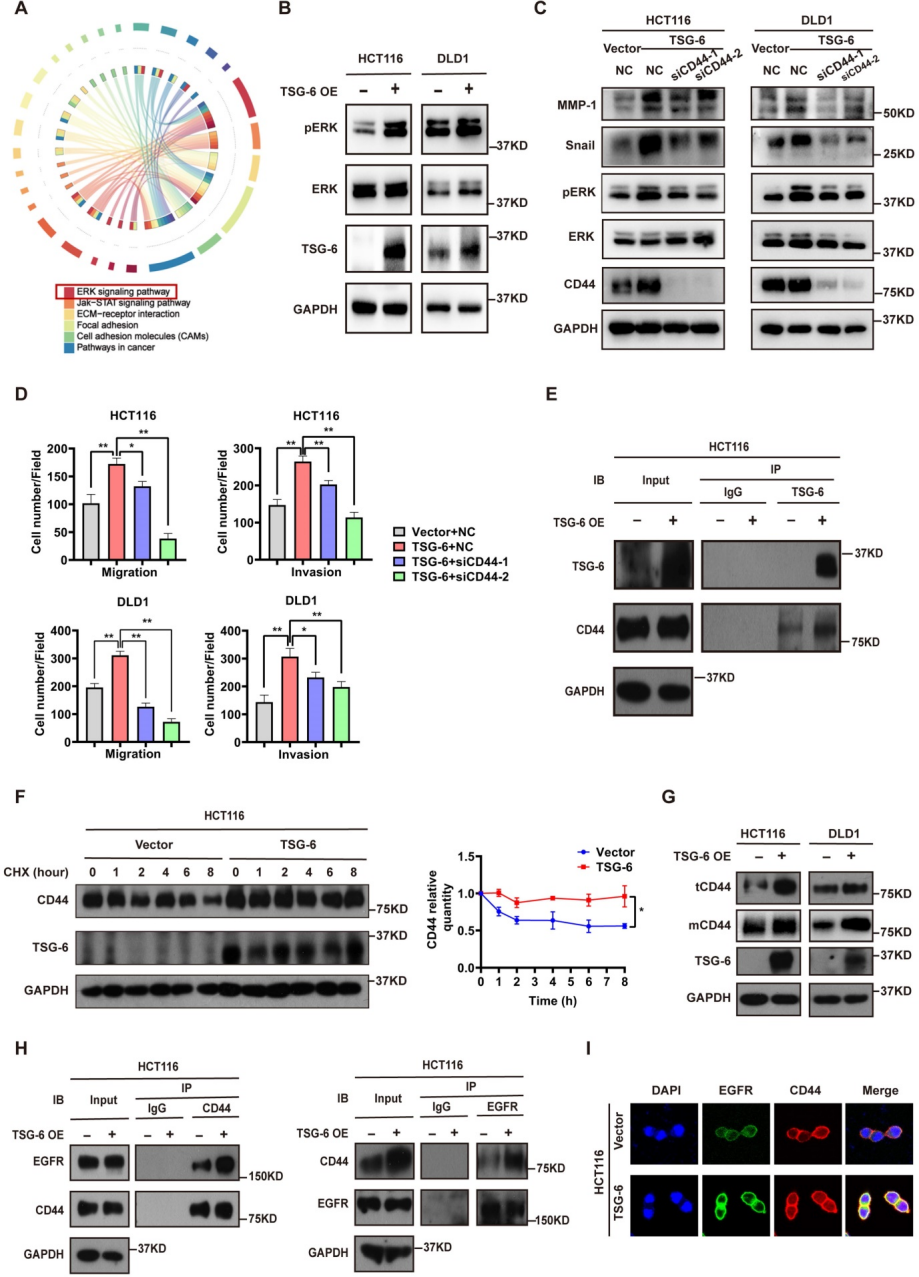
** TSG-6 promotes metastasis of CRC cells by facilitating cell membrane CD44-EGFR complex formation and downstream ERK activation. (A)** Chord diagram of KEGG results for mRNA expression profiles of TSG-6-overexpressed HCT116 cells versus control cells. TOP 1000 upregulated gene were selected to analyze. **(B)** Western blot evaluation of ERK phosphorylation in TSG-6-overexpressed CRC cells. **(C)** Western blot evaluation of indicated EMT markers and ERK phosphorylation in CRC cells co-transfected with TSG-6 overexpressing plasmids and siCD44. **(D)** Transwell migration and Matrigel invasion assays in CRC cells co-transfected with TSG-6 overexpressing plasmids and siCD44. Cell numbers shown in the bar chart were the average of five random fields. **(E)** CO-IP assay to determine the interaction between TSG-6 and CD44. **(F)** CHX chase assays to determine the half-life of CD44 in TSG-6-overexpressed HCT116 cells. **(G)** Western blot evaluation of total CD44 (tCD44) and membrane CD44 (mCD44) protein level in CRC cells. **(H)** CO-IP assay to determine the interaction between CD44 and EGFR upon TSG-6 overexpression. **(I)** Immunofluorescent analysis to investigate the co-localization of EGFR (green) and CD44 (red) in HCT116 transiently transfected with TSG-6 overexpressing plasmids. Data information: CRC cells used for the experiments were transiently transfected with plasmids or siRNA. Statistical analysis was performed using two tailed unpaired Student's t-test (D, F). *p<0.05, **p<0.01.

To further identify the critical intracellular signaling pathways associated with TSG-6 overexpression in CRC, we generated mRNA expression profiles from control samples and TSG-6 overexpressing HCT116 cells. TSG-6 overexpressed CRC cells exhibited activated ERK signaling (Figure 3A and S3A), which has been previously reported to be closely associated with the EMT process 20, 21. Subsequently, western blot analysis was used to validate the increased ERK protein phosphorylation levels in HCT116 and DLD1 cells as a consequence of TSG-6 overexpression (Figure 3B), or rhTSG-6 treatment (Figure S3B).

Studies have demonstrated that TSG-6 can modulate the interaction between hyaluronan (HA) and CD44 by inducing a HA conformational change 14, 15. Of note, CD44 has been linked to tumor metastasis, therapy resistance, and poor outcomes in various tumors 14, 22. For this reason, we first investigated whether TSG-6 can induce downstream ERK signaling activation and exert its pro-metastatic action depending on CD44 levels. Our findings demonstrated that TSG-6 induced ERK phosphorylation (Thr202/Tyr204) and EMT marker increase (Snail and MMP-1) could be suppressed by CD44 downregulation (Figure 3C and S3C). Moreover, TSG-6 enhanced cell migration and invasion were significantly inhibited by siCD44 (Figure 3D and S3D). Co-immunoprecipitation assays showed an interaction between TSG-6 and CD44 (Figure 3E). Furthermore, TSG-6 overexpression resulted in an increase in the CD44 protein level; however, it did not impact CD44 mRNA levels in CRC cells (Figure S3E and S3F). Cycloheximide (CHX) assays indicated that TSG-6 overexpression could enhance the endogenous CD44 protein's half-life (Figure 3F and S3G). Additionally, membrane protein extraction assays showed that TSG-6 overexpression resulted in an increase in both total and membrane CD44 levels in CRC cells (Figure 3G).

CD44 is a transmembrane protein that can form a complex with EGFR on the cell membrane to initiate downstream ERK activation 23, 24. Thus, we speculated whether TSG-6 can interact with CD44, and therefore affect the membrane CD44-EGFR complex in CRC cells. Overall, we found that TSG-6 overexpression could enhance the CD44-EGFR interaction in CRC cells (Figure 3H). Consistently, the immunofluorescence staining further confirmed that TSG-6 could facilitate the co-localization of CD44 and EGFR on the cell membrane (Figure 3I and S3H). Therefore, the previously noted results imply that TSG-6 may activate ERK signaling by stabilizing CD44 and facilitating the formation of the CD44-EGFR complex on the cell membrane.

To validate the association between TSG-6 and CD44 *in vivo*, TSG-6-stable overexpressed HCT116 (HCT116^TSG-6^) and control HCT116 (HCT116^Vector^) cells were injected into the mice spleens. IHC liver metastatic tumor tissue staining showed an increased CD44 level in the HCT116^TSG-6^ group (Figure S3I). Interestingly, expression of α-smooth muscle actin (α-SMA), a CAFs marker, in fibroblasts was also increased in the HCT116^TSG-6^ group (Figure S3I). Consequently, this observation indicated a possible role for TSG-6 in regulating the TME, especially regarding fibroblast activation, which extended our findings from cancer cells to TME fibroblasts.

### CRC Cells Derived TSG-6 Reprograms NFs into CAFs

**Figure 4:**
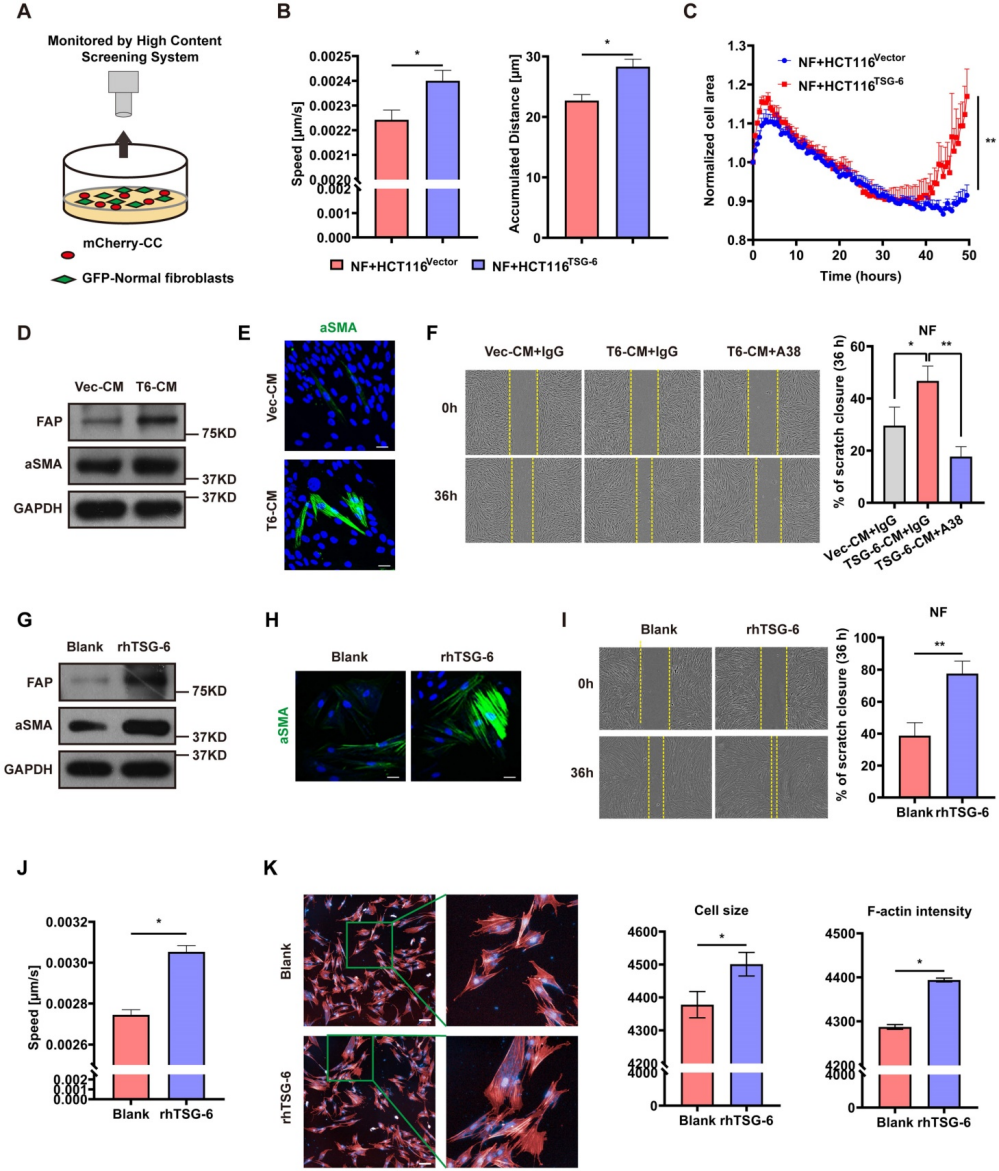
** CRC cells derived TSG-6 reprograms NFs into CAFs. (A)** Schematic representation of co-culture of cancer cells and GFP-labeled normal fibroblasts, cancer cells were transfected by mCherry-Vector plasmids or mCherry-TSG-6 overexpressing plasmids. **(B-C)** High Content Screening assay to monitor the speed, movement distance (B) and cell size (C) of NFs coculturing with CRC cells. **(D)** Western blot evaluation of α-SMA and FAP expression in NFs treated with Vec-CM or T6-CM. **(E)** Immunostaining of α-SMA in NFs treated with Vec-CM or T6-CM, detected by confocal microscopy (Magnification: 200×). Scale bars: 10 µm. **(F)** Wound healing assay to determine the migration of NFs cultured with T6-CM and Vec-CM. TSG-6 neutralizing antibody A38 could abolish the effect of T6-CM. **(G)** Western blot evaluation of α-SMA and FAP expression in NFs treated with or without rhTSG-6. **(H)** Immunostaining of α-SMA in NFs treated with or without rhTSG-6, detected by confocal microscopy (Magnification: 200×). Scale bars: 10 µm. **(I)** Wound healing assay to determine the migration of NFs treated with or without rhTSG-6. **(J-K)** High Content Screening assay to monitor the movement speed (J), cell size and F-actin intensity (K) of NFs treated with rhTSG-6. F-actin stained with phalloidin (red) and nuclei stained with DAPI (blue). Scale bars: 20 µm. Data information: Statistical analysis was performed using two tailed unpaired Student's t-test (B, C, F, I, J, K). *p<0.05, **p<0.01.

Given that TSG-6 was strongly associated with ECM remodeling (Figure S2A) and that α-SMA was increased in mice injected with TSG-6 overexpressed CRC cells (Figure S3I), we assessed whether TSG-6 overexpression in CRC cells could impact fibroblast activation via the paracrine effect. Primary fibroblasts were isolated from the normal colon mucosa of five CRC patients and pooled for future experiments. mCherry-labeled HCT116 cells were cocultured with GFP-labeled NFs and then monitored by the High Content Screening System (Figure 4A). NFs cocultured with TSG-6-overexpressed HCT116 cells displayed a more aggressive phenotype with enhanced migration and a larger tumor size than that with control cells (Figure 4B and 4C) as demonstrated 25, 26.

To mimic the paracrine secretion of TSG-6 by CRC cells, conditioned medium (CM) from TSG-6 overexpression cancer cells (T6-CM) or control cells (Vec-CM) was collected for the subsequent treatment of NFs. The T6-CM application increased α-SMA and fibroblast activation protein (FAP) levels (the CAFs typical markers) in NFs (Figure 4D). Immunofluorescence further confirmed the increased expression of α-SMA (Figure 4E). Significantly, T6-CM enhanced NFs migration could be attenuated by the TSG-6 neutralizing antibody A38 (Figure 4F). Thus, this finding indicates that the effect of T6-CM might be due to TSG-6 secretion into the TME. Similarly, rhTSG-6 (100 ng/ml) treatment could also increase the α-SMA, FAP, movement speed, cell size, and F-actin levels in NFs (Figure 4G-4K). The above results suggest that TSG-6 overexpressed CRC cells may transform NFs to CAFs by TSG-6-induced paracrine activation.

### TSG-6 triggers paracrine activation of JAK2-STAT3 signaling in NFs

**Figure 5:**
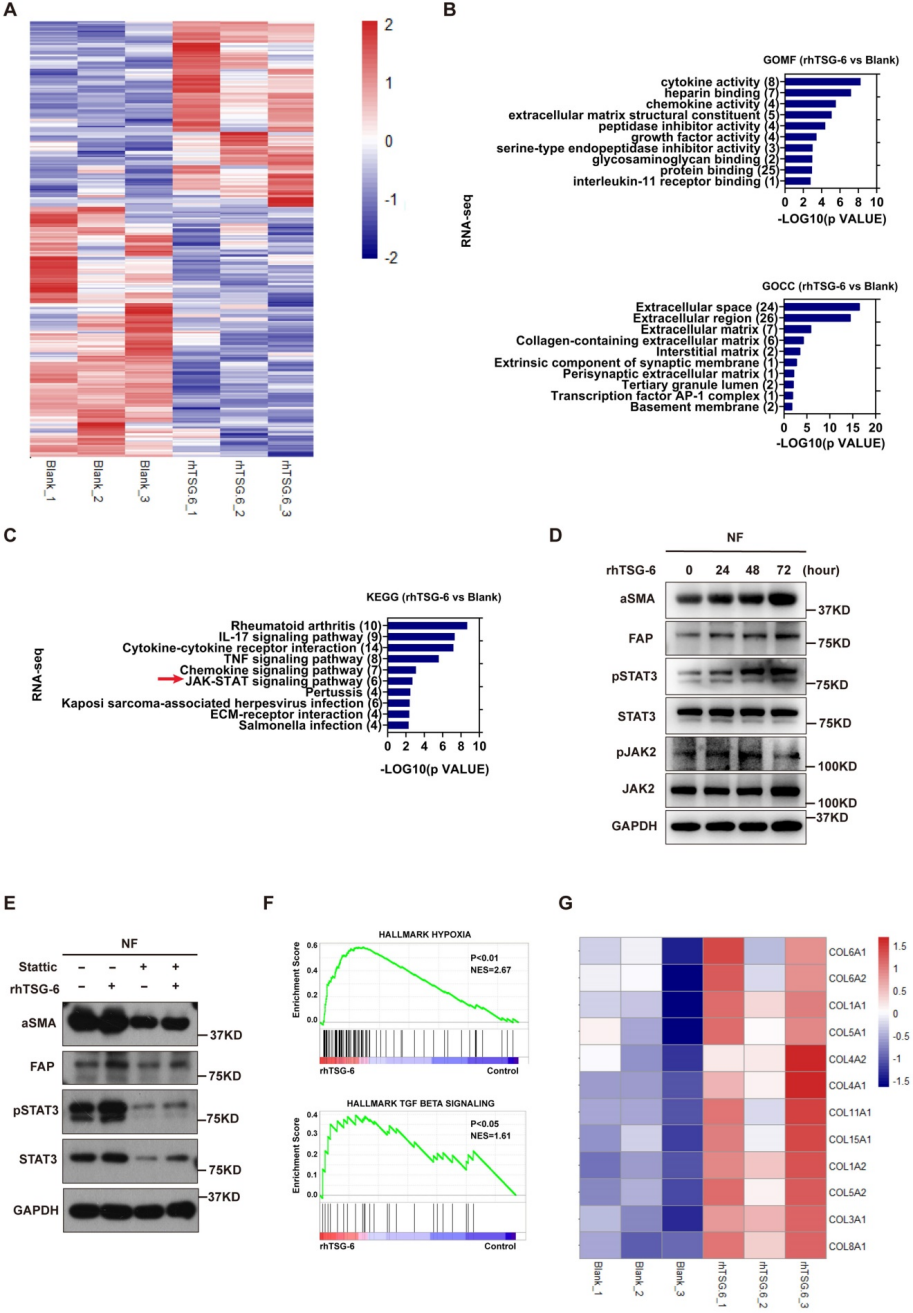
** TSG-6 triggers paracrine activation of JAK2-STAT3 signaling in NFs. (A)** Heat map displays dysregulated genes (p<0.05, |log2 FC|≥0.5) in fibroblasts after rhTSG-6 treatment (detail genes in supplementary Table S1). **(B)** GO analysis of the TOP 275 dysregulated gene for RNAseq identified enrichment of ECM remodeling and cytokine secretion features in rhTSG-6 treated NFs versus control NFs. GOMF means GO molecular function; GOCC means GO cellular component. **(C)** KEGG analysis for RNAseq showing the enrichment of JAK-STAT signaling pathway in rhTSG-6 treated NFs versus control NFs. **(D)** Western blot evaluation of indicated markers in NFs treated with rhTSG-6 for 0, 24, 48, 72 hours. **(E)** Western blot evaluation of indicated markers in NFs pre-treated with STAT3 inhibitor (Stattic) for 6 hours and then cultured with rhTSG-6 for 24 hours. **(F)** GSEA results showing enrichment of HALLMARK (hypoxia and TGF-b signaling) in NFs treated with rhTSG-6. **(G)** Heat map showing collagen expression in NFs upon rhTSG-6 treatment.

To further characterize the NFs' gene expression signature activated by TSG-6 overexpressed cancer cells, we performed Human Cytokine Antibody Arrays (440) on NFs treated with CRC cell-derived condition medium and rhTSG-6. Both cell lysates and culture supernatants were collected and analyzed. KEGG analyses showed enrichment of the JAK-STAT signaling pathway (Figure S4A and S4B), which has been associated with fibroblast activation 27, 28. Considering the complexity of the condition medium component, RNA-seq was performed on NFs treated with rhTSG-6 to identify the gene expression signature directly associated with TSG-6 stimulation (Figure 5A and S4C-S4D).

GSEA, KEGG and GO analyses were used to predict the functional role of the differentially expressed genes. GO identified upregulated ECM remodeling and cytokine secretion features in rhTSG-6 treated NFs (Figure 5B), which are also the characteristics of CAFs. KEGG analysis also indicated that rhTSG-6 treated NFs were enriched for JAK-STAT signaling (Figure 5C). To verify this relevance, NFs were stimulated with rhTSG-6 for different time periods (0h, 24h, 48h, and 72h). Moreover, the pJAK2 (Tyr-1007/1008) and pSTAT3 (Tyr-705) levels were increased, together with α-SMA and FAP upregulation in a time-dependent manner (Figure 5D). Subsequently, these features could be abolished by pre-treatment with STAT3 specific inhibitor Stattic (5 μM) for 6h (Figure 5E).

Furthermore, GSEA analysis also indicated that TSG-6-induced gene expression in NFs was positively correlated with hypoxia and TGF-β signaling (Figure 5F). Notably, we observed that multiple collagen types were upregulated in NFs following rhTSG-6 treatment (Figure 5G). Studies have shown that CAFs can form a collagen-rich TME, thereby creating a stiff ECM, which compresses intratumoral blood vessels and aggravates hypoxia 29, 30. Collectively, TSG-6 may turn NFs into CAFs through JAK2-STAT3 signaling pathway activation, thus remodeling the ECM into a stiff and hypoxic TME.

### TSG-6-overexpressed cancer cells and fibroblasts drives metastasis *in vivo*

**Figure 6:**
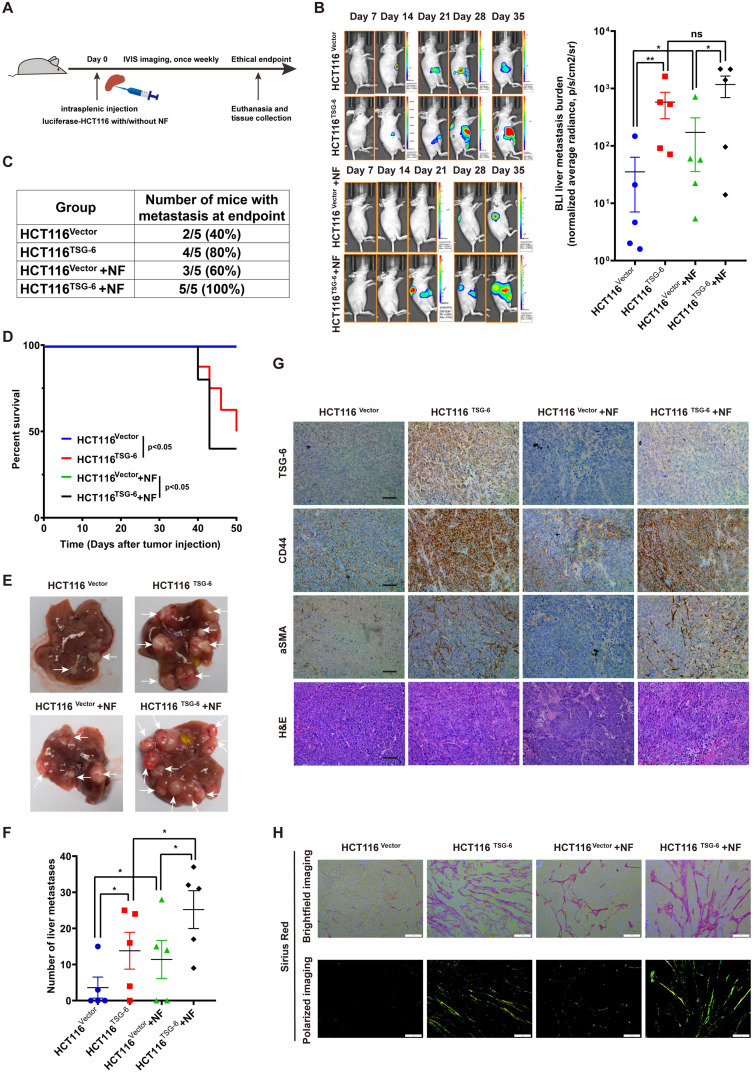
** Crosstalk between TSG-6-overexpressed cancer cells and normal fibroblasts drives metastasis *in vivo*. (A)** Schematic representation of intrasplenic injection of TSG-6-overexpressed HCT116 with or without fibroblasts and IVIS monitoring of liver metastasis. **(B)** Left panel: representative images of whole body IVIS imaging in mice bearing liver metastatic burden from four groups, five mice for each group (n=5). Right panel: statistical chart summarized the average bioluminescent imaging signal of all mice at the end of the experiment for each group. **(C)** Table summarizing liver metastatic rate for each group. **(D)** Kaplan-Meier analysis of survival in mice for each group. **(E-F)** Representative images of metastatic foci (pointed out with white arrow) in mice liver (E) and number of liver metastatic foci for mice (F) in all four groups. **(G)** IHC staining of TSG-6, CD44, α-SMA and H&E staining in paraffin-embedded sections from the resected mice livers. Scale bar represent 100 µm. **(H)** Representative images of picrosirius red staining imaged with brightfield or polarized light. Scale bar represent 100 µm. Data information: Error bars represent mean ± S.E.M. Statistical analysis was performed using two tailed unpaired Student's t-test (B, F) and Log-rank Gehan-Breslow-Wilcoxon test (D). *p<0.05, **p<0.01.

To investigate the impact of TSG-6 on CRC metastasis *in vivo,* we injected HCT116 cells stably overexpressing luciferase and TSG-6 (HCT116^TSG-6^) into the spleen of nude mice. The control group was injected with HCT116 overexpressing luciferase (HCT116^Vector^) (Figure 6A). TSG-6-overexpressing tumors showed a stronger BLI signal and a higher liver metastatic rate, worse survival, and more metastatic foci in the liver than in the control group (Figure 6B-6F).

Meanwhile, we evaluated the synergistic effects of fibroblasts and TSG-6-overexpressed cancer cells on metastasis by intrasplenic co-injections of luciferase-labeled HCT116^TSG-6^ /HCT116^Vector^ cells and fibroblasts, as described elsewhere 31, 32. HCT116^TSG-6^ cells coinjected with NFs (HCT116^TSG-6^ +NF) had a higher liver metastatic rate, worse survival, and more liver metastatic foci than HCT116^Vector^ cells coinjected with NFs (HCT116^Vector^ +NFs group) (Figure 6C-6F). Notably, the HCT116^TSG-6^ +NF group showed the highest liver metastatic rate, worst survival rate, and most liver metastatic foci among the four groups. Similarly, we also noted the higher manifestation of ascites occurred higher in the HCT116^TSG-6^ +NF group than in all the other groups. Consistent with our *in vitro* studies, IHC staining of mice liver tissue showed that TSG-6 could increase CD44 and α-SMA expression in cancer cells and fibroblasts, respectively (Figure 6G).

Picrosirius red staining indicated a significant increase of fibrillar collagen density in the HCT116^TSG-6^ and HCT116^TSG-6^ +NF groups (Figure 6H), demonstrating that TSG-6 could remodel ECM at metastatic sites by activating NFs, thus forming a collagen-rich TME. These results indicate that TSG-6 can promote cancer cell metastasis *in vivo*. More importantly, co-injection of fibroblasts may contribute to a pro-metastatic TME that facilitates cancer cell inoculation and migration.

### TSG-6 activated CAFs promote tumor progression

**Figure 7:**
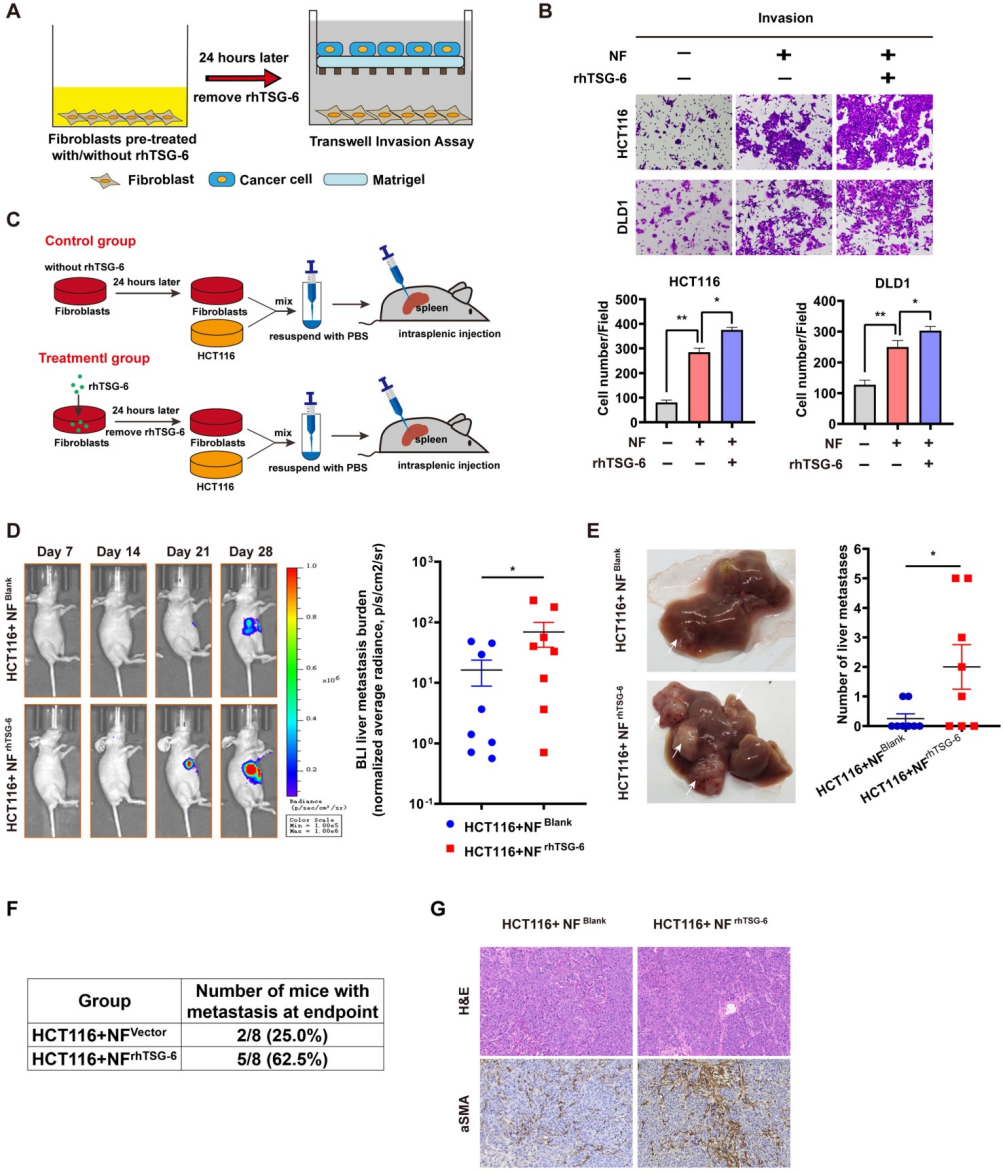
** TSG-6 activated CAFs in turn promotes CRC metastasis. (A)** Schematic representation of Transwell Invasion Assay. Normal fibroblasts were pretreated with or without rhTSG-6 for 24 hours and then changed to normal culture medium. Cancer cells and fibroblasts were seeded in the upper wells and lower wells, respectively. **(B)** Representative image of transwell invasion assays. Cell numbers shown in the bar chart were the average of five random fields. **(C)** Schematic representation of intrasplenic injection of HCT116 with normal fibroblasts which were pretreated with or without rhTSG-6 for 24 hours. IVIS monitoring of liver metastasis was performed once weekly. **(D)** Left panel: representative images of whole body IVIS imaging in mice bearing liver metastatic burden from both groups, eight mice for each group (n=8). Right panel: statistical chart summarized the average bioluminescent imaging signal of all mice at the end of the experiment for each group. **(E)** Representative images of metastatic foci (pointed out with white arrow) in mice liver and number of liver metastatic foci for mice in both groups. **(F)** Table summarizing liver metastatic rate for both groups. **(G)** IHC staining of α-SMA and H&E staining in paraffin-embedded sections from the resected mice livers for both groups. Scale bar represent 100 µm. Data information: Error bars represent mean ± S.E.M. Statistical analysis was performed using two tailed unpaired Student's t-test (B, D, E). *p<0.05, **p<0.01.

Studies have definitively established that activated CAFs can promote tumor progression by secreting large quantities of proteins, including MMPs, growth factors, proinflammatory cytokines, and chemokines, into the TME 33. To enhance our understanding of how TSG-6-activated fibroblasts affect cancer cells, we analyzed the results of the Human Cytokine Antibody Arrays of fibroblasts treated with rhTSG-6 or CRC cell derived condition medium. Culture supernatant GO analyses showed an upregulated secretion of cytokine, chemokine, and growth factor in rhTSG-6 or T6-CM treated NFs (Figure S5A), with a significant upregulation of CCL5 (also termed RANTES) and MMP-3 secretion (Figure S5B and S5C). CCL5 and MMP-3 are considered vital pro-metastatic factors secreted by CAFs 34-38. Transwell assays further confirmed that rhTSG-6 pretreated NFs could promote CRC cell invasion (Figure 7A-B).

We further investigated the pro-metastatic effect of TSG-6-activated NFs *in vivo*. NFs pretreated with/without rhTSG-6 (NF^rhTSG-6^ and NF^Blank^) for 24h were coinjected with HCT116 into the mice spleen (Figure 7C). The NF^rhTSG-6^ group showed stronger BLI signals, higher liver metastatic rates, and more metastatic foci in the liver (Figure 7D-7F). Mice liver tissue IHC staining showed an increase in CAFs marker α-SMA levels in fibroblasts (Figure 7G). Collectively, the above results demonstrate that CRC cells derived from TSG-6 can transform NFs into the CAF phenotype, which may in turn result in the secretion of large quantities of pro-metastatic factors into the TME, thereby enhancing the metastatic ability of cancer cells.

## Discussion

TSG-6 has been widely studied in many diseases that are closely related to the inflammation process, including rheumatoid arthritis 39, 40, inflammatory bowel disease 41, 42, systemic lupus erythematosus 43 and so on. To date, only a few studies have mentioned the involvement of TSG-6 in malignant disorders 12, 44. The underlying TSG-6 molecular mechanism in cancer has never been addressed. Herein, we determined the oncogenic role of TSG-6 in CRC. Our findings established the upregulation of TSG-6 in CRC tumors, especially in the most aggressive CMS4 CRC. Moreover, higher TSG-6 levels were significantly associated with advanced CRC grade and unfavorable prognosis. Furthermore, TSG-6 overexpression promoted CRC metastasis both *in vitro* and *in vivo* and exerted an important role in CAFs activation and CRC progression.

Abnormal ECM stiffness has been reported to play a crucial role in cancer progression and metastasis 45, 46. Studies have shown that HA reorganization is strongly associated with ECM remodeling and stiffness 47. TSG-6 can affect the interaction between HA and CD44, thereby inducing ECM remodeling 15, 16. Our data showed that TSG-6 could enhance the CD44 half-life, resulting in its upregulation on the cell membrane. Moreover, we found an interaction between TSG-6, CD44, and EGFR, which is crucial for ERK signaling activation. As ERK signaling activation plays a vital role in many cancers 23, 48, our results revealed that TSG-6 may promote CRC metastasis by activating ERK signaling and EMT. Furthermore, TSG-6 may potentially impact CD44-EGFR complex formation by binding HA and modulating ECM remodeling. Thus, it would be interesting to further investigate whether TSG-6 overexpression in CRC cells can induce ECM stiffness by regulating HA reorganization, therefore facilitating cancer metastasis.

Fibroblasts are the most abundant stromal cells in the TME. Many studies have demonstrated the vital role of fibroblasts in cancer development. However, the function of fibroblasts remains controversial. The interplay between cancer cells and the TME fibroblasts is highly complex and heterogeneous. How they interact with each other under different situations and different TMEs is not fully elucidated. It has been reported that NFs can be educated by cancer cells and transformed into CAFs to enable their pro-metastatic ability 49, 50. Activated CAFs can subsequently affect cancer cells by secreting multiple growth factors, cytokines, and chemokines into the ECM 51. Furthermore, our results indicate that tumor-derived TSG-6 could transform NFs into CAFs by activating JAK2-STAT3 signaling. This effect could be abolished by a TSG-6 neutralizing antibody, implying that TSG-6 overexpressed CRC cells may secrete TSG-6 into the ECM and act on TME fibroblasts. In addition, a positive correlation was established between TSG-6-induced gene expression in NFs and TGF-β signaling. Activated TGF-β signaling is reported as one of the major causes of CAFs activation 52. As the CMS4 CRC is characterized by elevated TGF-β signaling 53, the presence of TSG-6-activated fibroblasts may significantly contribute to the CMS4 signature. Moreover, CAFs activated by TSG-6 could secrete pro-metastatic factors or chemokines such as MMP-3 and CCL5 in return 34, 38. Nonetheless, there are some limitations to our study. The sample size was insufficient, and clinical samples from multiple centers are needed to further verify the clinical significance of TSG-6 in CRC patients. Besides, as a secretory protein, the TSG-6 plasma level in CRC patients could be measured to explore its potential clinical value in prognosis and prediction of metastasis. A TSG-6 neutralizing antibody could be used in animal models to investigate its ability to block CRC metastasis *in vivo*. Moreover, further research is necessary to explore the detailed molecular mechanism by which TSG-6 reprograms NFs into CAFs and the way in which TSG-6-activated CAFs promote CRC metastasis in return.

**Figure 8:**
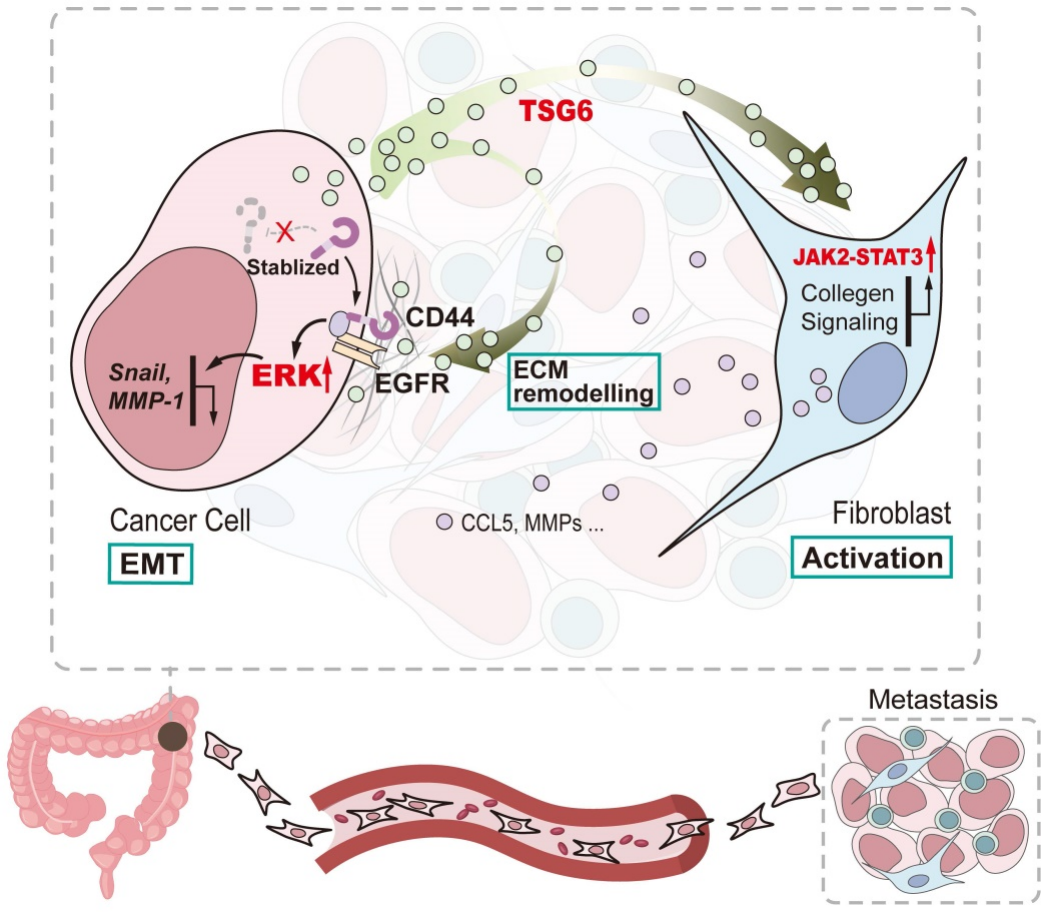
Schematic diagram of TSG-6 creating a pro-metastatic microenvironment in CRC. TSG-6-CD44-ERK autocrine signaling pathway in CRC cells and TSG-6-JAK2-STAT3 paracrine signaling pathway in fibroblasts synergistically promote CRC metastasis.

In summary, our study shows that TSG-6 overexpression in CRC could promote tumor metastasis and is significantly associated with poor clinical outcome. Mechanistically, TSG-6-induced EMT depends on the TSG-6-CD44-ERK axis in cancer cells in an autocrine manner; however, TSG-6 derived from CRC cells activates CAFs via JAK2-STAT3 signaling in a paracrine manner (Figure 8). Our study unraveled a TME-mediated mechanism for CRC metastasis. Therefore, targeting TSG-6 might be a potential therapeutic strategy for mCRC, which has important clinical implications.

## Supplementary Material

Supplementary figures, tables 1-2, materials and methods.

Supplementary table 3.

## Figures and Tables

**Table 1:** Correlation between expression of TSG-6 and clinicopathological features in CRC patients

Variables	Low TSG-6 (n=120)	High TSG-6 (n=86)	*P* value
**Gender**			0.001**
Male	80(66.7%)	37(43.0%)	
Female	40(33.3%)	49(57.0%)	
**Median age**			0.85
≤67 years	63(52.5%)	44(51.2%)	
>67 years	57(47.5%)	42(48.8%)	
**pT stage**			0.057
T1	8(6.7%)	7(8.1%)	
T2	29(24.2%)	18(20.9%)	
T3	76(63.3%)	46(53.5%)	
T4	7(5.8%)	15(17.4%)	
**pN stage**			<0.0001***
N0	98(81.7%)	46(53.5%)	
N1	19(15.8%)	31(36.0%)	
N2	3(2.5%)	9(10.5%)	
**pM stage**			0.002**
M0	112(93.3%)	68(79.1%)	
M1	8(6.7%)	18(20.9%)	
**TNM stage**			<0.0001***
I	36(30.0%)	24(27.9%)	
II	59(49.2%)	20(23.3%)	
III	17(14.2%)	24(27.9%)	
IV	8(6.6%)	18(20.9%)	
**Histological grade**			0.069
G1	45(37.5%)	27(31.4%)	
G2	73(60.8%)	52(60.5%)	
G3	2(1.7%)	7(8.1%)	

All data are shown as numbers and percentages. TSG-6 Low is defined as IHC score≤4 and TSG-6 High is defined as IHC score>4. *p<0.05, **p<0.01, ***p<0.001.

**Table 2:** Univariate and multivariate analysis of different parameters for Overall Survival in CRC patients

Variables	Univariate analysis		Multivariate analysis	
	HR (95%CI)	*p* value	HR (95%CI)	*p* value
Gender (male vs female)	1.170 (0.691-1.980)	0.559		
Age ( >67 vs ≤67)	1.721 (1.017-2.913)	0.043*		
Clinical stage (III-IV vs I-II)	8.542 (4.785-15.248)	<0.001***	7.690 (4.248-13.921)	<0.001***
Histological grade (G3 vs G1-G2)	7.586 (3.407-16.888)	<0.001***	2.719 (1.205-6.138)	0.016*
Recurrence (YES vs NO)	2.411 (0.962-6.041)	0.06		
TSG-6 expression (High vs Low)	1.970 (1.174-3.307)	0.010*		

Univariate and multivariate Cox proportional hazards regression were used to calculate Hazard ratio (HR), 95% confidence intervals (95% CI) and p values in SPSS 24.0. TSG-6 Low is defined as IHC score≤4 and TSG-6 High is defined as IHC score >4. *p<0.05, **p<0.01, ***p<0.001.

**Table 3:** Univariate and multivariate analysis of different parameters for Progression-Free Survival in CRC patients

Variables	Univariate analysis		Multivariate analysis	
	HR (95%CI)	*P* value	HR (95%CI)	*P* value
Gender (male vs female)	1.132 (0.689-1.859)	0.625		
Age ( >67 vs ≤67)	1.474 (0.902-2.409)	0.121		
Clinical stage (III-IV vs I-II)	6.886 (4.078-11.627)	<0.001***	5.722 (3.317-9.871)	<0.001***
Histological grade (G3 vs G1-G2)	6.451 (2.920-14.251)	<0.001***	2.846 (1.253-6.465)	0.012*
Recurrence (YES vs NO)	5.803 (2.731-12.332)	<0.001***	4.155 (1.922-8.981)	<0.001***
TSG-6 expression (High vs Low)	1.750 (1.075-2.849)	0.024*		

Univariate and multivariate Cox proportional hazards regression were used to calculate Hazard ratio (HR), 95% confidence intervals (95% CI) and p values in SPSS 24.0. TSG-6 Low is defined as IHC score≤4 and TSG-6 High is defined as IHC score>4. *p<0.05, **p<0.01, ***p<0.001.

## Abbreviations

CRC: colorectal cancer
TME: tumor microenvironment
TSG-6: tumor necrosis factor α stimulated gene 6
ECM: extracellular matrix
RT-qPCR: Real-Time Quantitative Reverse Transcription PCR
NFs: normal fibroblasts
CAFs: cancer-associated fibroblasts
95% CI: 95% confidence interval
HR: Hazard ratio
SYSU: Sun Yat-sen University
