# Supplementary material for: TSG-6 promotes Cancer Cell aggressiveness in a CD44-Dependent Manner and Reprograms Normal Fibroblasts to create a Pro-metastatic Microenvironment in Colorectal Cancer
Source: Int J Biol Sci. 2022 Feb 7;18(4):1677–94. doi: 10.7150/ijbs.69178 (PMC8898369; doi:10.7150/ijbs.69178)
Supplement: Supplementary file 1 — Supplementary figures, tables 1-2, materials and methods. [file ijbsv18p1677s1.pdf]

Fig S1

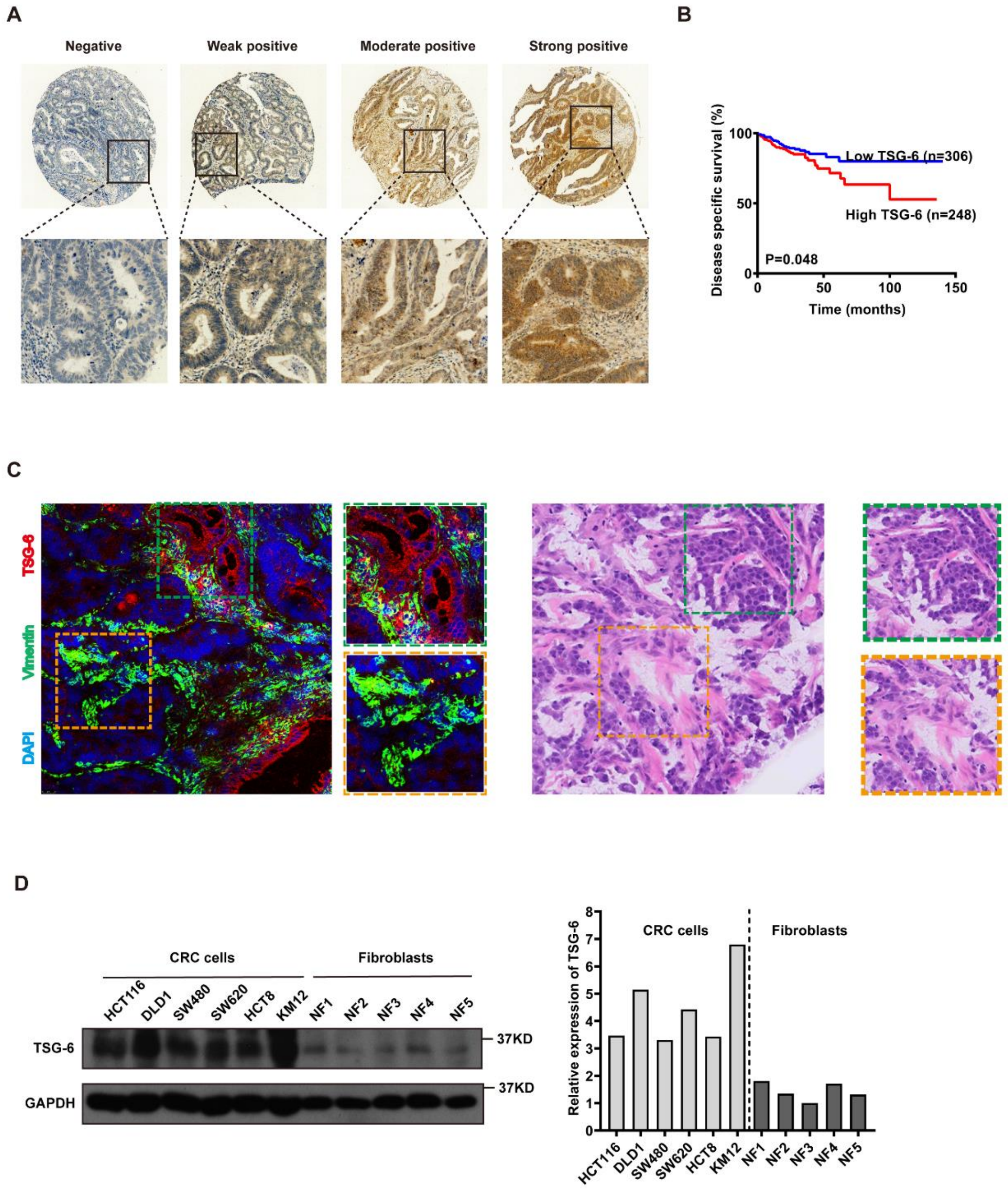

Figure S1. TSG-6 is highly expressed in CRC cells.

(A) Representative TSG-6 IHC staining on human CRC TMAs.

(B) DSS of CRC patients (TCGA), stratified by TSG-6 mRNA expression, Log-rank Gehan-Breslow-Wilcoxon test.

(C) Left panel: representative images of TSG-6 (Red) and Vimentin (Green) co-IF staining of colon cancer tissue counterstained with DAPI (Blue). Right panel:

corresponding H&E staining of colon cancer tissue. Green and orange squares show central and tumor-adjacent areas, respectively.

(D) Left panel: western blot analysis of TSG-6 in CRC cell lines and normal fibroblasts. Right panel: relative quantity of WB results.

Fig S2

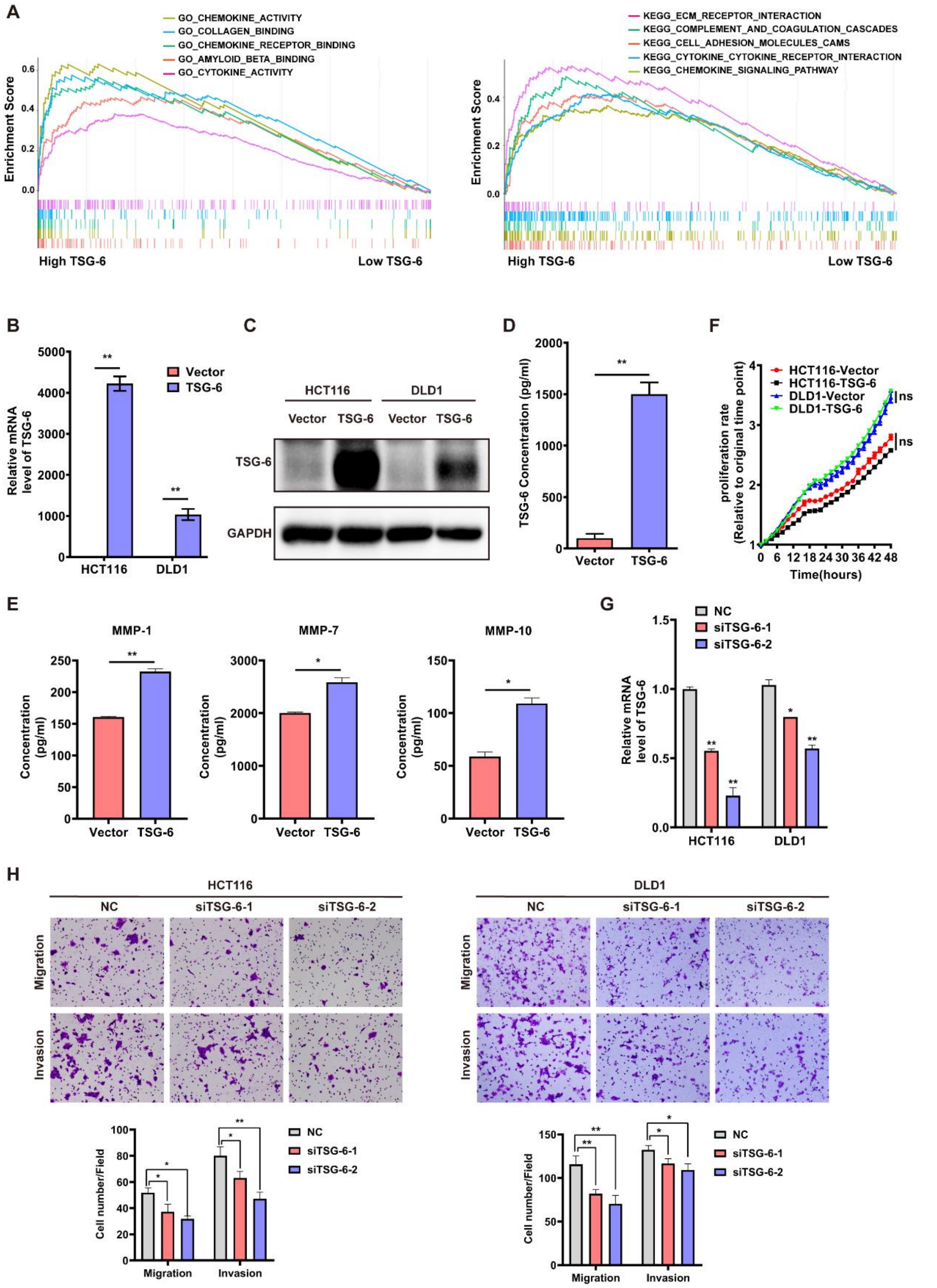

Figure S2. TSG-6 promotes migration and invasion by inducing EMT in CRC cells.

(A) GSEA results showing GO and KEGG using GSE14333 datasets.

(B-C) TSG-6 overexpressed in HCT116 and DLD1 via plasmid transfection, detected

by RT-qPCR (B) and western blot (C).

(D) TSG-6 protein level in supernatant of HCT116 transfected with TSG-6 overexpressed plasmid or vector, detected by ELISA.

(E) Multiplex immunoassay was performed to determine levels of MMP-1, MMP-7 and MMP-10 secreted by TSG-6-overexpressed HCT116.

(F) Proliferation assays performed in CRC cells transiently overexpressing TSG-6.

(G) TSG-6 silencing in HCT116 and DLD1 via transfected with siRNA, detected by RT-qPCR.

(H) Transwell migration and invasion assays were performed to compare cell motilities in HCT116 and DLD1 cells transfected with TSG-6 siRNA. Cell numbers shown in the bar chart were the average of five random fields.

Data information: Error bars represent mean ± S.E.M. Statistical analysis was performed using two tailed unpaired Student's t-test (B, D, E, F, G, H). *$p<0.05$, **$p<0.01$

Fig S3

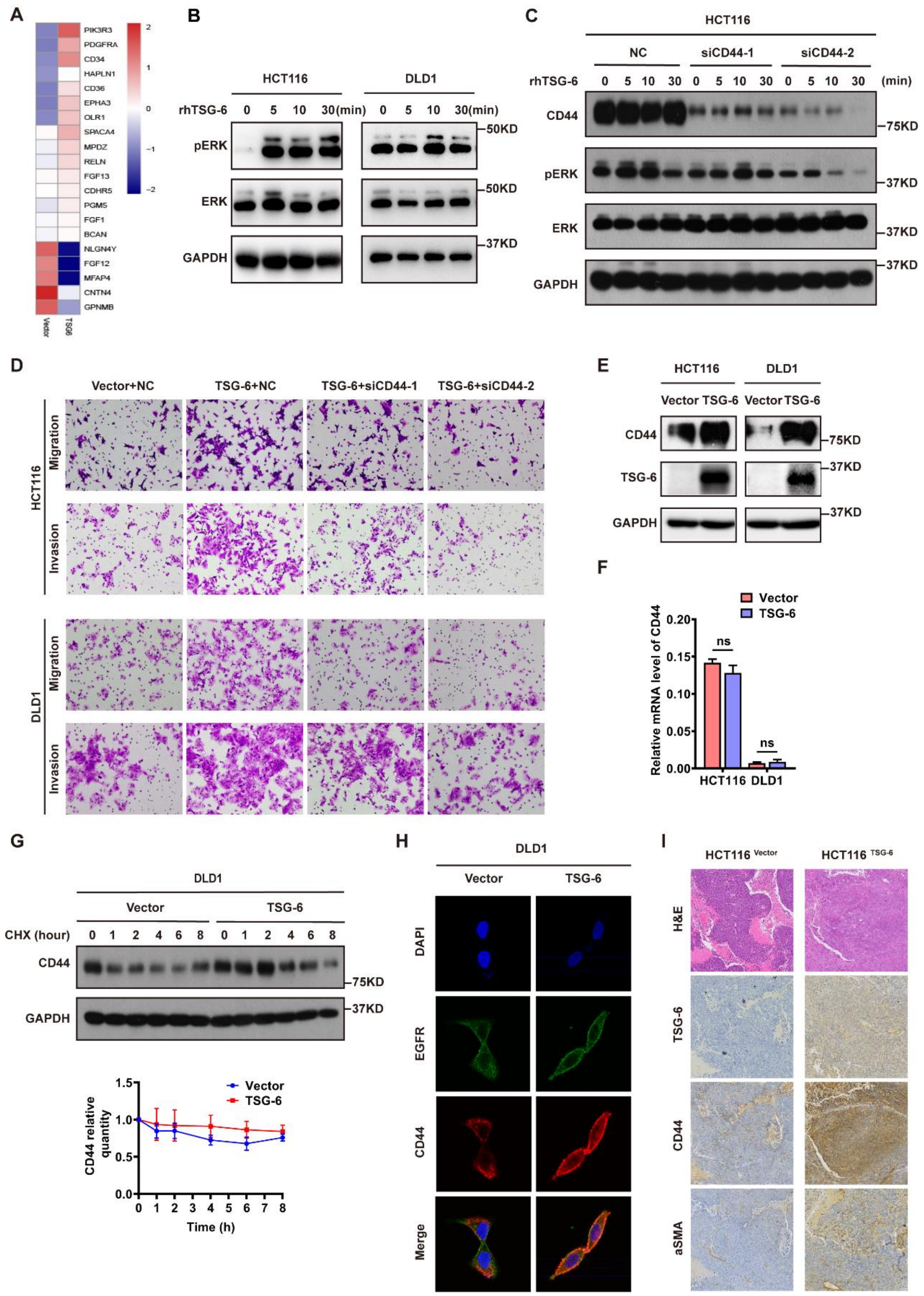

Fig S3. TSG-6 promotes metastasis of CRC cells by facilitating cell membrane CD44-EGFR complex formation and downstream ERK activation.

(A) Heatmap of EKR marker expression in control and TSG-6-overexpressed HCT116

cells.

(B) Western blot evaluation of ERK (Thr202/Tyr204) phosphorylation in CRC cells treated by rhTSG-6.

(**C)** Western blot evaluation of ERK (Thr202/Tyr204) phosphorylation in HCT116 cells induced by rhTSG-6 treatment after knockdown of CD44 with siRNA.

(**D)** Transwell migration and Matrigel invasion assays in CRC cells after co-transfection with TSG-6 overexpressing plasmids and CD44 siRNA.

(**E-F)** CD44 level in CRC cells transfected with control or TSG-6 overexpressing plasmids, detected by western blot (E) and RT-qPCR (F).

(**G)** CHX chase assays to determine the half-life of CD44 in TSG-6-overexpressed DLD1 cells.

(**H)** Immunofluorescent analysis to investigate the co-localization of EGFR (green) and CD44 (red) in DLD1 transiently transfected with TSG-6 overexpressing plasmids.

(**I)** IHC staining of TSG-6, CD44, α-SMA and H&E staining in paraffin-embedded sections from the resected mice livers for both groups, three mice each group (n=3).

Data information: Error bars represent mean ± S.E.M. Statistical analysis was performed using two tailed unpaired Student's t-test (F, G). *p<0.05, **p<0.01

Fig S4

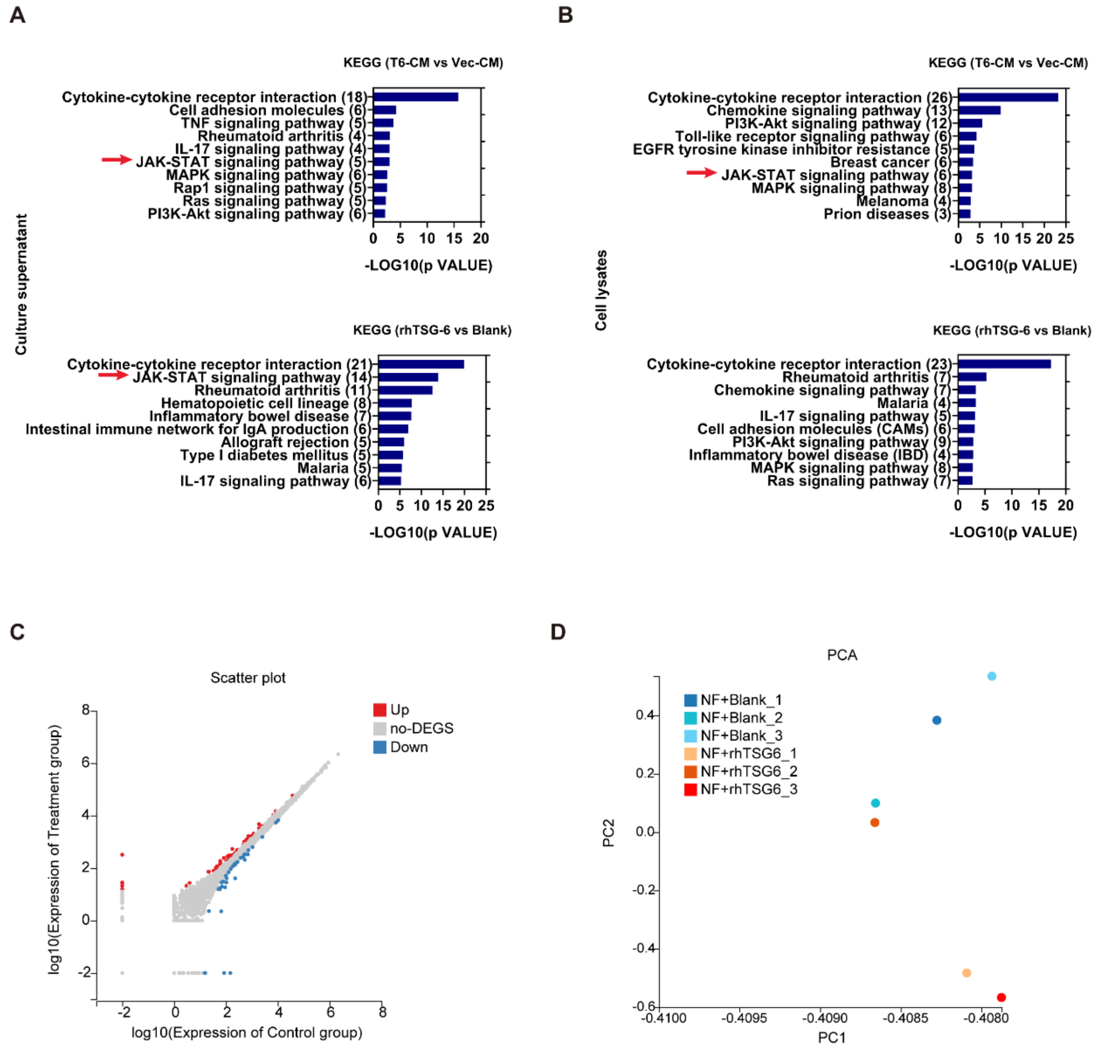

Fig S4. TSG-6 triggers paracrine activation of JAK2-STAT3 signaling in NFs

(A) KEGG analyses for human Cytokine Antibody Arrays of culture supernatant of fibroblasts treated with T6-CM or rhTSG-6.

(B) KEGG analyses for human Cytokine Antibody Arrays of cell lysates of fibroblasts treated with T6-CM or rhTSG-6.

(C) The scatter plot of differentially expressed genes in fibroblasts treated with or without rhTSG-6, Up = upregulated genes, Down = downregulated genes, no-DEGS = no differentially expressed genes.

(D) The principal component analysis based on gene expression of fibroblasts treated with or without rhTSG-6.

Fig S5

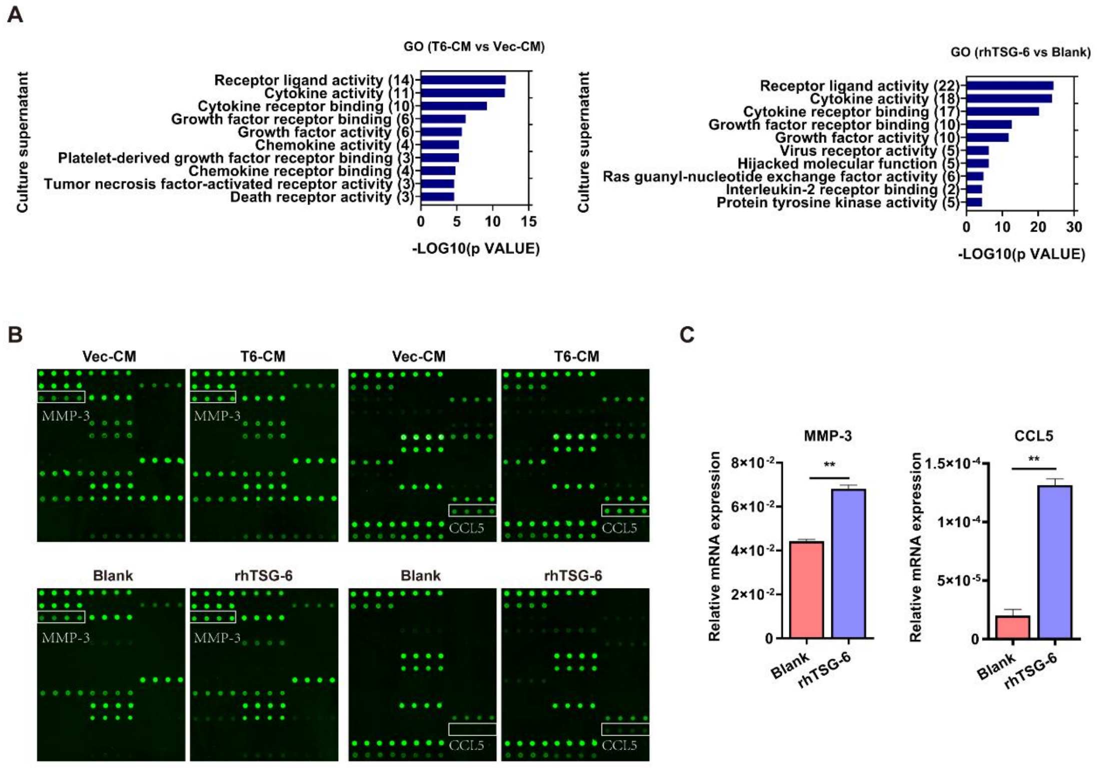

Fig S5. TSG-6 activated CAFs in turn promotes CRC metastasis.

(A) GO analyses for human Cytokine Antibody Arrays of culture supernatant of fibroblasts treated with T6-CM or rhTSG-6.

(B) Human Cytokine Antibody Arrays results indicated a significant upregulated secretion of MMP-3 and CCL5 in NFs after T6-CM or rhTSG-6 treatment.

(C) RT-qPCR was performed to determined mRNA level of MMP-3 and CCL5 in normal fibroblasts after treatment with rhTSG-6. Error bars represent mean ± S.E.M. Statistical analysis was performed using two tailed unpaired Student's t-test. *p<0.05, **p<0.01

Supplementary tables

Table S1. Antibodies used in this study

Name	Company	Catalog Number	Assay
GAPDH	Proteintech	60004-1-Ig	WB
TSG-6	Santa Cruz Biotech	sc-377277	WB, IHC, IF
TSG-6	Santa Cruz Biotech	sc-65886	Neutralizing antibody
E-cadherin	Cell signaling technology	#3195	WB
β-catenin	BD Transduction Laboratories	#610154	WB
Snail	Cell signaling technology	#3895	WB
MMP-1	Millipore	Mab13439	WB
ERK	Cell signaling technology	#4695	WB
P-ERK (Thr202/Tyr204)	Cell signaling technology	#4370	WB
CD44	Cell signaling technology	#3570	WB, IHC, IF, IP
EGFR	Cell signaling technology	#4267	WB, IF
αSMA	Cell signaling technology	#19245	IF
αSMA	Santa Cruz Biotech	sc-32251	WB, IHC
FAP	Abcam	ab53066	WB
JAK2	Cell signaling technology	#3230	WB
P-JAK2 (Tyr1007/1008)	Cell signaling technology	#3771	WB
STAT3	Cell signaling technology	#9139	WB
P-STAT3 (Tyr705)	Cell signaling technology	#9145	WB
Mouse IgG1 Isotype Control	R&D System	MAB002	Control for IP, neutralizing antibody

Table S2 Primers and siRNA sequence used in this study

**Primers used for qRT-PCR**		
**Name**	**Forward-primer**	**Reverse-primer**
GAPDH	GACAGTCAGCCGCATCTTCTT	AATCCGTTGACTCCGACCTTC
TSG-6	GATGCCTATTGCTACAACCCAC	GGTGAATACGCTGACCATACTTGA
MMP-3	GGAGACTTTTACCCTTTTGATGG	TGGTCCCTGTTGTATCCTTTGT
CD44	CTGCCGCTTTGCAGGTGTA	CATTGTGGGCAAGGTGCTATT
CCL5	TGACCTGTGGACGACTGC	TGCTTTGCCTACATTGCC
IL-1a	GCCCAAGATGAAGACCAACCAGT	CCGTGAGTTTCCCAGAAGAAGAGG
IL-4	CGGCAGTTCTACAGCCACCAT	GCCGTTTCAGGAATCGGATCA
IL-11	AGCTGAGGGACAAATTCC	CGCAGGTAGGACAGTAGGT
**Primers used for plasmid construction**		
**Name**	**Forward-primer**	**Reverse-primer**
pcDNA 3.1(+)-TSG-6	CAGTGTGGTGGAATTGGGCGCGC ATGATCATCTTAATTTACTTATTTC	GATATCTGCAGAATTCTTTATAAG TGGCTAAATCTTCCAGC
PCDH-TSG-6	CATAGAAGATTCTAGATGATCAT CTTAATTTACTTATTTC	ATTCGCTAGCTCTAGTTATAAGTG GCTAAATCTTCC

Sequence for siRNA	
TSG-6-siRNA1	GGCTGATTATGTTGAAATA
TSG-6-siRNA2	GCTGGAGGTTTCCAAATCA
CD44-siRNA1	GCAGTCAACAGTCGAAGAA
CD44-siRNA2	CTCTGAGCATCGGATTTGA

Supplementary materials and methods

1. RNA extraction and quantitative real-time polymerase chain reaction (qRT-PCR)

Total RNA was extracted from frozen tissue samples or CRC cell lines by using TRIzol reagent (Invitrogen) according to manufacturer's protocol and was reversely transcribed as cDNA with ReverTra Ace qPCR RT kit (Toyobo, Japan) immediately. TSG-6 mRNA levels were quantified by qPCR analysis which was performed with FastStart Essential DNA Green Master (Rhoche, Germany) in LightCycler 96 Instrument. The primers used in this study are listed in supplementary table 2.

2. Western blot analysis

Total proteins were extracted from cultured cells by T-PER tissue protein extraction reagent (Thermo, Rockford, IL, USA) with phosphatase inhibitor cocktail set II and protease inhibitor cocktail set III (Millipore, Germany) following manufacturer's instructions. The membrane proteins from cultured cells were extracted by Membrane Protein Extraction Kit (BestBio, Shanghai, China) according to the manufacturer's instructions. The protein extracts were separated by sodium dodecyl sulfate-polyacrylamide gel electrophoresis (SDS-PAGE). The proteins separated on SDS-PAGE were transferred onto a PVDF membrane (Millipore, San Diego, CA, USA). The membrane was blocked for 1 hour in Tris-Buffered saline and Tween 20 (TBST) with 4% BSA. And the proteins on PVDF membrane was probed with primary antibodies

and followed by incubation with appropriate secondary antibodies. Proteins were detected by ECL chemiluminescence system (Millipore) on autoradiography film (Kodak, Rochester, NY, USA) or ChemiDoc Touch (Bio-Rad).

3. Cell proliferation, migration and invasion assay

For cell proliferation assay, CRC cells were transfected by TSG-6 overexpression plasmid or control vector, then plated in 96-well plates (five thousand cells per well) 48 hours later. Cell proliferation process were captured by Incucyte Zoom System (Essen Bioscience, USA), photographed every four hours. The average percentage of cell confluence per group at each time point was calculated and proliferation curve was performed by GraphPad Prism 8.0.

For migration and invasion assay, 24-well Boyden chambers with 8-μm pore size (Corning, NY, USA) were inserted in a 24-well plate precoated with Matrigel (cell invasion assay) or without Matrigel (cell migration assay). HCT116 (1.0×10^5) or DLD1 (4.0×10^5) were suspended in 200μl serum-free culture medium and placed into the upper chamber per filter. 600μl culture medium supplemented with 20% FBS was placed into the lower chamber and incubated at 37°C for 36 hours (for DLD1) or 48 hours (for HCT116). The migrated cells were fixed in 4% paraformaldehyde for 15 minutes at room temperature, stained with 0.1% crystal violet for 5 minutes, and imaged five fields per chamber under microscopy (Olympus, Japan). Migrated cells were counted by ImageJ. For promoting invasion of HCT116 or DLD1 by rhTSG-6-induced CAFs, 2.0×10^4 normal fibroblasts were placed into the lower chamber and pretreated with/without 100ng/ml rhTSG-6 for 24 hours, then replaced the culture medium with 600μl culture medium supplemented with 20% FBS. 24-well Boyden chambers with 8-μm pore size (Corning, NY, USA) were inserted in a 24-well plate precoated with Matrigel. HCT116 (1.0×10^5) or DLD1 (4.0×10^5) were suspended in 200μl serum-free culture medium, then placed into the upper chamber and incubated at 37°C for 36 hours (for DLD1) or 48 hours (for HCT116). The rest of steps are the same as above. The experiments were repeated three times and the average number of cells per field were calculated.

4. Enzyme-Linked Immunosorbent Assay and Multiplex Immunoassay

TSG-6 level in cell culture supernatant was detected using and Human TSG-6 enzyme-linked immunosorbent assay (ELISA) kit (RayBiotech, GA, USA) according to the manufacturer's instructions. The minimum detectable dose of Human TSG-6 was determined to be 200 pg/ml.

For multiplex immunoassay, MMPs secreted from HCT116 transfected with TSG-6 overexpressing plasmids for 72 hours were measured by Human MMP Panel 2 (Milliplex Map Kit, Millipore, USA).

5. Co-immunoprecipitation (co-IP)

HCT116 cells transfected with TSG-6 or Vector for 48 hours were lysed in 600μl IP lysis buffer for 30 minutes on ice. Cell lysates were centrifugated at 12,000g for 10 minutes at 4℃ and the protein supernatant was collected, 60 μl supernatant was used as input. Then the remaining protein supernatant was incubated with Mouse IgG1 Isotype Control (R&D System, MAB002) or Anti-CD44 (CST, #3570) overnight at 4℃. Then the mixture was incubated with the Sepharose-conjugated protein G magnetic beads (Thermo Fisher Scientific, MA, USA) overnight at 4℃. After washing with IP wash buffer for four times, the beads were centrifugated and mixed with 1x loading buffer and boiled for 10 minutes. The protein of co-IP and input samples were determined by western blot analysis.

6. Immunofluorescence

HCT116 and DLD1 were harvested after transfection with indicated plasmids (pcDNA 3.1(+)-TSG-6 or pcDNA 3.1(+)-Vector) for 48 hours, and then plated on the coverslips. For staining of CD44 and EGFR, cells were fixed with 4% paraformaldehyde for 20 minutes at room temperature, permeabilized with 0.1% Triton X-100 for 5 minutes, then blocked with 1% bovine serum albumin (BSA) for 45 minutes. Then cells were incubated simultaneously with both anti-CD44 (1:400) and anti-EGFR (1:100) at 4℃ overnight. The following day, cells were simultaneously incubated with both goat anti-rabbit Alexa Fluor 488 conjugated secondary antibody and goat anti-mouse Alexa Fluor

542 conjugated secondary antibody (Invitrogen, USA) at room temperature for 1 hour, followed by staining cell nuclei with DAPI (Sigma Aldrich, Germany). The images were captured by Leica TCS-SP8 confocal microscopy (Mannheim, Germany). For αSMA staining in fibroblasts, cells were grown on glass coverslips and treated with rhTSG-6 (100ng/ml) for 0, 24, 48, 72 hours. Immunofluorescence staining was performed by using anti-αSMA (1:200, CST, USA) as primary antibody, detected via goat anti-rabbit Alexa Fluor 488 conjugated secondary antibody. Other procedures were the same as above. For TSG-6 and αSMA staining in frozen sections of tumor tissue from CRC patients, the sections were exposed to room temperature for twenty minutes and then washed by PBS for ten minutes to remove optimal cutting temperature (OCT) compound. Next, the sections were permeabilized with 0.5% Triton X-100 for 20 minutes, then blocked with 3% bovine serum albumin (BSA) for 1 hour. Then the sections were incubated simultaneously with both anti-TSG-6 (1:100) and anti-αSMA (1:100) at 4℃ overnight. The remaining steps were the same as above.
